# NAT10‐mediated ac^4^C modification promotes ectoderm differentiation of human embryonic stem cells via acetylating *NR2F1* mRNA

**DOI:** 10.1111/cpr.13577

**Published:** 2023-12-02

**Authors:** Junbang Ge, Zhaoxia Wang, Ji Wu

**Affiliations:** ^1^ Bio‐X Institutes, Key Laboratory for the Genetics of Developmental and Neuropsychiatric Disorders, Ministry of Education Shanghai Jiao Tong University Shanghai China; ^2^ Laboratory Animal Center of Instrumental Analysis Center Shanghai Jiao Tong University Shanghai China; ^3^ Key Laboratory of Fertility Preservation and Maintenance of Ministry of Education, School of Basic Medical Sciences Ningxia Medical University Yinchuan China; ^4^ Shanghai Key Laboratory of Reproductive Medicine Shanghai China

## Abstract

Cell fate determination in mammalian development is complex and precisely controlled and accumulating evidence indicates that epigenetic mechanisms are crucially involved. *N* ^4^‐acetylcytidine (ac^4^C) is a recently identified modification of messenger RNA (mRNA); however, its functions are still elusive in mammalian. Here, we show that N‐acetyltransferase 10 (NAT10)‐mediated ac^4^C modification promotes ectoderm differentiation of human embryonic stem cells (hESCs) by acetylating nuclear receptor subfamily 2 group F member 1 (*NR2F1*) mRNA to enhance translation efficiency (TE). Acetylated RNA immunoprecipitation sequencing (acRIP‐seq) revealed that levels of ac^4^C modification were higher in ectodermal neuroepithelial progenitor (NEP) cells than in hESCs or mesoendoderm cells. In addition, integrated analysis of acRIP‐seq and ribosome profiling sequencing revealed that NAT10 catalysed ac^4^C modification to improve TE in NEP cells. RIP‐qRT‐PCR analysis identified an interaction between NAT10 and *NR2F1* mRNA in NEP cells and NR2F1 accelerated the nucleus‐to‐cytoplasm translocation of yes‐associated protein 1, which contributed to ectodermal differentiation of hESCs. Collectively, these findings point out the novel regulatory role of ac^4^C modification in the early ectodermal differentiation of hESCs and will provide a new strategy for the treatment of neuroectodermal defects diseases.

## Graphical abstract

NAT10 catalysed ac^4^C modification to improve translation efficiency in ectodermal cells. In addition, the NAT10 interacted with nuclear receptor subfamily 2 group F member 1 (*NR2F1*) mRNA, which could increase its stability and translation, thereby promoting the expression of NR2F1. This increase in NR2F1 expression could accelerate the nuclear export of yes‐associated protein 1 (YAP1), which facilitated ectodermal differentiation of hESCs.

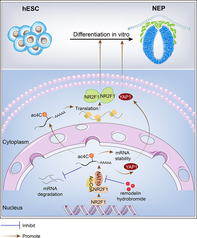

## 1 INTRODUCTION

Significant lineage diversification, cell destiny specification, and tissue patterning occur throughout the early stages of human embryonic development. Early human embryonic development remains largely unknown because human embryo samples are difficult to obtain, and interspecies variation makes extrapolating results from animal studies challenging. Human embryonic stem cells (hESCs) are a great model for modelling early human embryonic development because they exhibit unrestricted proliferative capacity and multidirectional differentiation in vitro.^1^

Human embryonic development is complex yet requires precise execution. The main challenge is to accurately explain how complex regulatory elements interact to regulate the expression of a single gene. Messenger RNA (mRNA) carries genetic information into the cytoplasm and combines with ribosomes. After processing and translation, protein molecules are synthesised that have particular spatial structures. Dysregulation of mRNA translation leads to multiple diseases, such as amyotrophic lateral sclerosis^2^ and Alzheimer's disease.^3^ Therefore, it is important to understand how cells integrate the varied information encoded by nucleotide sequences to regulate mRNAs. The extent to which regulation of translation contributes to the dynamic transformation of cells, such as in stem cell differentiation, is still poorly understood.

Accumulating evidence shows that the integration of cellular events depends on epigenetic marks.^4^, ^5^, ^6^ *N* ^4^‐acetylcytidine (ac^4^C), a modification of the mRNA, has recently been recognised as an important factor in post‐transcriptional regulation.^7^, ^8^ Moreover, the different effects that ac^4^C mediates on mRNA translation are position‐dependent. The stability and translation of the target mRNA can be enhanced when ac^4^C is broadly distributed in a region with coding sequences; however, when ac^4^C is present in the 5′‐untranslated region (5′‐UTR), it facilitates upstream initiation.^9^

The sole known ac^4^C writer in mammals, N‐acetyltransferase 10 (NAT10), catalyses the acetylation of cytidine residues in mRNA. NAT10 is emerging as a crucial regulator in certain cancers, including bladder,^10^ pancreatic^11^ and colon^12^ cancers. To promote viral replication, NAT10 additionally inserts ac^4^C to many distinct sites on human immunodeficiency virus type 1 (HIV‐1) transcripts.^13^ In testes and ovaries, NAT10 is enriched and displays dynamic changes from the late zygotene stage to the round spermatid stage in spermatogenesis.^14^ However, the function and mechanism of NAT10‐mediated mRNA acetylation in germ layer differentiation of hESCs are known little.

In the current study, we found that ectodermal cells expressed NAT10 at higher levels than in mesoendoderm (ME) cells or hESCs. Using acetylated RNA immunoprecipitation sequencing (acRIP‐seq), we further observed ac^4^C modification to be higher in ectodermal cells compared with hESCs or ME cells. Furthermore, NAT10 catalysed ac^4^C modification to improve translation efficiency (TE) in ectodermal cells. In addition, the NAT10 interacted with nuclear receptor subfamily 2 group F member 1 (*NR2F1*) mRNA, which could increase its stability and translation, thereby promoting the expression of NR2F1. This increase in NR2F1 expression could accelerate the nuclear export of yes‐associated protein 1 (YAP1), which facilitated ectodermal differentiation of hESCs.

## 2 MATERIALS AND METHODS

### 2.1 hESC differentiation

Differentiation of hESCs into neuroepithelial progenitor (NEP) cells was performed using a previously reported method with minor modification.^15^ In brief, hESCs were dissociated with dispase II and seeded on mitomycin C‐treated mouse embryonic fibroblast cells in six‐well plates. The number of feeder cells and hESCs were approximately 3 × 10^5^ cells and 8 × 10^4^ cells per well, respectively. On the following day (Day 1), the hESC medium was switched to NEP medium, consisting of DMEM/F12 and neurobasal medium (1:1) with 0.1 mM ascorbic acid (A4403; Sigma), 1X N2 supplement (17502048; Gibco), 1X B27 supplement (12587010; Gibco), 3 μM CHIR99021 (SML1046; Sigma), 1% GlutaMax™ (35050061; Gibco), 2 μM DMH1(HY‐12273; MCE) and 2 μM SB431542 (HY‐10431; MCE) and it took 6 days to culture the cells.

Differentiation of hESCs into ME cells was performed according to prior work.^16^ The number of feeder cells and hESCs was approximately 1 × 10^5^ and 8 × 10^4^ per well, respectively, in six‐well plates. The hESC medium was changed to advanced RPMI1640 (12633012; Gibco), supplemented with 1X penicillin/streptomycin (15070063; Gibco), CHIR99021 (5 μM, SML1046; Sigma) and 1% GlutaMax™ (35050061; Gibco).

### 2.2 RNA dot blotting

RNA dot blotting was conducted using an anti‐ac^4^C antibody, as previously described, with few adjustments.^14^, ^17^ Briefly, TRIzol was used to separate the RNA from cells. The Dynabeads™ mRNA Purification Kit was used to purify the mRNA and RNA was placed in denaturation solution (20X SSC solution: RNA = 1:1) at 95°C for 5 min, then quickly placed on ice for 2 min. RNA was then added to DEPC‐treated Amersham Hybond‐N+ membranes. Membranes were crosslinked six times at 150 mJ/cm^2^ in a UV 254 nm crosslinker and then immersed into methylene blue stain for 5 min. The stained membranes were photographed. Membranes were washed in 75% ethanol for 2 min and blocked for 1 h. Then, the membranes were incubated with an anti‐ac^4^C antibody (1:1000; Abcam) for 2 h at 25°C. Membranes were incubated with an HRP‐conjugated secondary antibody (1:2000; ProteinTech) for 1.5 h. After incubating the Super ECL Detection Reagent (Yeasen Biotechnology, Shanghai, China), the membranes were visualised using a FUSION FX imager (VILBER, France).

### 2.3 acRIP‐seq and acRIP‐qRT‐PCR

Flow cytometric sorting was used to harvest the hESCs, NEP and ME cells for acRIP‐seq. The CloudSeq Inc. (Shanghai, China) completed the acRIP‐seq. The procedure of acRIP‐seq was conducted as previously described.^18^ In short, total RNA was isolated from each cell. NEBNext rRNA Depletion Kit v2 was used to remove rRNA and the GenSeq® ac^4^C‐IP Kit (GS‐ET‐005, GenSeq Inc., China) was used to immuneprecipitate RNA. Briefly, RNA Fragmentation Reagents were used to fragment the RNA to ~200 nt. The ac^4^C antibody was added to the Protein A/G beads for 1 h. The RNA fragments/antibody complexes were incubated for 4 h at 4°C. Then, the complexes were treated with Proteinase K and phenol: chloroform was used to purify the RNA. The RNA was subsequently used for acRIP‐qRT‐PCR or construction of the RNA libraries. An Agilent 2100 bioanalyzer was used to qualify the RNA Libraries and sequencing was performed on a NovaSeq platform (Illumina). Primers used for quantitative reverse transcription‐polymerase chain reaction (qRT‐PCR) were described in Table S1.

### 2.4 RNA immunoprecipitation assay

Approximately 1 × 10^7^ cells were harvested for RNA immunoprecipitation (RIP) assay. The RNA Immunoprecipitation Kit (GENESEED, P0102) was used for RIP as directed by the manufacturer. In brief, cells were lysed in buffer A working solution for 10 min. Supernatants were obtained by centrifugation at 14,000 × *g*. One third of the supernatant was saved as input. Meanwhile, protein A + G beads were incubated with Buffer A and Buffer D at 4°C for 30 min. After washing twice, the magnetic beads were divided into two for incubation with RIP or IgG antibodies for 2 h, and then washed twice with Buffer A. The Buffer A and one third of the cell lysate supernatant were added to the magnetic beads bound with NAT10 RIP antibody or IgG, and incubated overnight at 4°C. Beads were then washed with Buffer B for 2 min, five times. The complexes bound to the magnetic beads were eluted with Buffer E. The input samples were similarly treated with Buffer E. The collected supernatants were added to DR Columns to remove DNA. The supernatants were collected by centrifugation at 14,000 × *g* for 2 min. Seventy percent ethanol was then added and the solution was loaded onto RC Columns. The RC filter columns were then washed using Buffer F and Buffer G by centrifugation at 12,000 × *g* for 1 min. To obtain the RNA, the filter columns were transferred to a new RNase‐free tube and an appropriate volume of RNase‐free water was added and centrifugation was performed at 12,000 × *g* for 5 min. RNA samples were subjected to reverse transcription using cDNA Synthesis SuperMix (Yeasen, Co., Ltd., Shanghai, China). Samples were quantified by qRT‐PCR, and normalised to the input and IgG group using the 2^−ΔΔ*Ct*^ approach.

### 2.5 RNA decay assay

The DMSO‐treated NEP cells (Day 7) and remodelin hydrobromide (RE)‐treated NEP cells (Day 7) were treated with mRNA transcription inhibitor DRB (75 μM, D1916; Sigma‐Aldrich, USA) for 0, 20 and 40 min. Then, total RNA was isolated and used to determine the levels of *NR2F1* mRNA (relative to 0 min) by qRT‐PCR. The half‐life of mRNA was estimated as prior described.^19^

### 2.6 Protein stability assay

The protein stability assay was according to the previous reports.^20^, ^21^, ^22^ Briefly, the DMSO‐treated NEP cells (Day 7) and RE‐treated NEP cells (Day 7) were treated with an inhibitor of protein synthesis, cycloheximide (CHX, 100 μg/mL; Sigma, USA). The cells were obtained at indicated times for analysing the protein stability of NR2F1 by Western blotting.

### 2.7 Puromycin intake assay

The puromycin intake assay was conducted according to the previous report.^23^, ^24^ In short, the DMSO‐treated hESCs‐derived NEP cells (Day 7) and RE‐treated hESCs‐derived NEP cells (Day 7) were incubated with puromycin (1 μg/mL; Beyotime, Shanghai, China) for 30 min at 37°C. RIPA lysis buffer was used to lyse the cells, and the same amount of protein was loaded for Western blotting.

### 2.8 Ribosome profiling sequencing

Ribosome profiling sequencing (Ribo‐seq) was completed by Gene Denovo Biotechnology Co. (Guangzhou, China). In brief, the hESCs, NEP and ME cells were treated with CHX (100 μg/mL; Sigma, USA) for 5 min to freeze the translating ribosomes. Cells were lysed with lysis buffer and centrifuged at 20,000 × *g* for 10 min at 4°C. RNase I (New England Biolabs, Inc.) and DNase I (New England Biolabs, Inc.) were added to the supernatant for 45 min at room temperature. SUPERase·In RNase inhibitor (Ambion, Austin, TX, USA) was added to stop the nuclease digestion. The RNA Clean and Concentrator‐25 kit (Zymo Research; R1017) was used to isolate RFs with a size greater than 17 nt. The rRNA was removed by adding short (50–80 base) antisense DNA probes complementary to rRNA sequences, RNase H (New England Biolabs, Inc.) and DNase I (New England Biolabs, Inc.). Magnetic beads (Vazyme, Nanjing, Jiangsu, China) were used to further purify the RFs. Libraries were created using the NEBNext® Multiple Small RNA Library Prep Set for Illumina® (E7300L) and sequenced using the Illumina HiSeq™ X10 (Gene Denovo Biotechnology Co.).

### 2.9 Statistical analysis

The Student *t*‐test was conducted for the compare groups. Analysis of variance was used for continuous variables. IBM SPSS Statistics 26 was used for statistical analysis. The significance level is shown in each graph as follows: *p < 0.05; **p < 0.01; ***p < 0.001; ^ns^ *p* > 0.05. Unless otherwise noted, each experiment was conducted thrice with biologically independent samples.

## 3 RESULTS

### 3.1 ac^4^C modification is highly enriched in hESC‐derived ectodermal cells

We first differentiated the hESCs into ME cells and ectodermal NEP cells (Figure S1A). The hESCs exhibited colony‐like growth and the hESC‐derived NEP cells displayed neural tube‐like structures. Flow cytometry sorting was performed to obtain the target cells (Figure S2A–C). The expression levels of various marker genes such as the octamer‐binding transcription factor4 (OCT4) in hESCs, homeobox A3 (HOXA3), sry‐box transcription factor 1 (SOX1) and neural cell adhesion molecule 1 (NCAM1) in NEP cells and t‐box transcription factor t (TBXT) and mix paired‐like homeobox (MIXL1) in ME cells were confirmed using RT‐PCR (Figure S1B), qRT‐PCR (Figure S1C–F) and immunofluorescence staining (Figure S1G).

**FIGURE 1:**
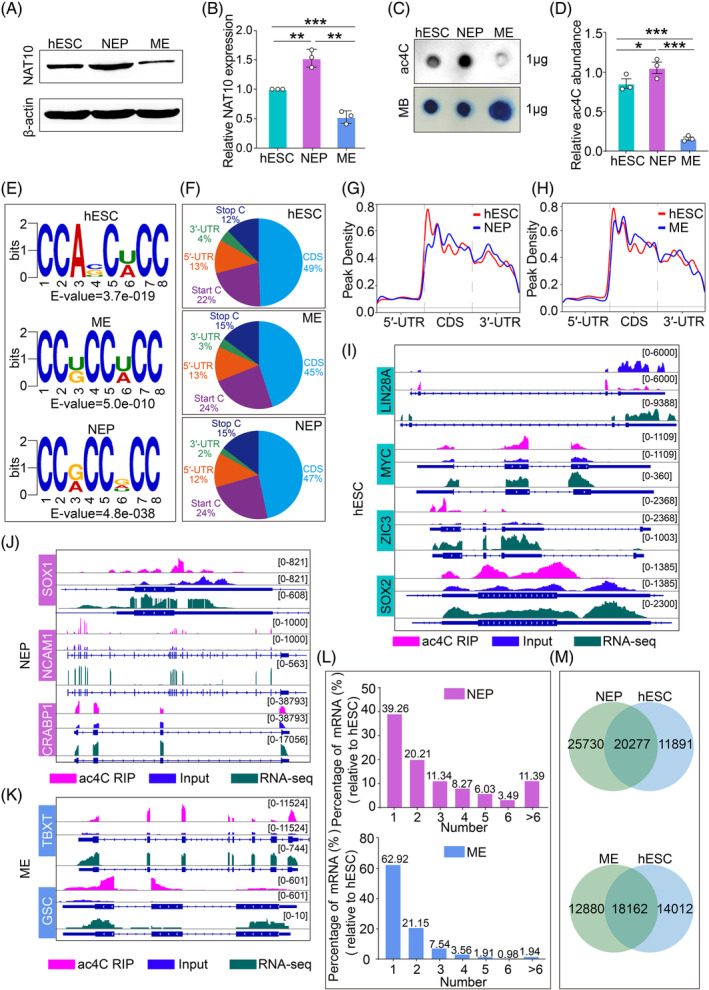
Detection of ac^4^C modifications in hESCs and hESC‐derived different germ layer cells. (A) NAT10 protein levels in hESCs, NEPs, and ME cells were measured using a Western blot method. (B) Quantification of relative NAT10 levels in (A). (C) Dot blot analysis of ac^4^C levels in 1 μg RNA from hESCs and hESC‐derived NEP and ME cells using an anti‐ac^4^C antibody. The internal standard was achieved using methylene blue (MB) staining. (D) Quantification of relative ac^4^C modification abundance in NEP and ME cells. (E) Sequence motifs enriched within ac^4^C peaks were identified by acRIP‐seq in hESCs, NEP and ME cells. (F) Distribution of ac^4^C peaks. (G, H) Peak density of the ac^4^C peaks across the mRNA transcripts in NEP cells versus hESCs (G) and ME cells versus hESCs (H). (I–K) Integrative Genomics Viewer (IGV) displayed the distribution of acRIP‐seq and RNA‐seq reads of pluripotency regulators (LIN28A, MYC, ZIC3 and SOX2) in hESCs (I), ectodermal NEP marker genes (SOX1, NCAM1 and CRABP1) in NEP cells (J) and mesoendodermal marker genes (TBXT and GSC) in ME cells (K). Pink reads were from anti‐ac^4^C IP libraries; blue reads were from input libraries; dark cyan reads were from RNA‐seq libraries. (L) The percentage of mRNAs in NEP (pink) and ME (blue) cells with varying amounts of ac^4^C peaks. (M) Numbers of differential ac^4^C peaks were detected for NEP cells versus hESCs and ME cells versus hESCs. Error bars indicate mean ± SEM.

To examine the level of ac^4^C modification during early hESC differentiation, the levels of the ac^4^C writer protein, NAT10, were analysed during in vitro differentiation of hESC. The results suggested that the protein levels of NAT10 were significantly increased after ectodermal differentiation (Figure 1A,B). RNA dot blotting (Figure 1C,D) further confirmed that ac^4^C modifications were more abundant in NEP cells than in hESCs or ME cells. These results indicated that NAT10‐mediated ac^4^C modification might exert a crucial role during ectodermal differentiation.

To further examine the role of ac^4^C mRNA modification in the early differentiation of hESCs, we determined global profiles of ac^4^C modification in hESCs, NEP and ME cells, using acRIP‐seq. De novo motif analysis identified the typical CXXCXXCX ac^4^C sequence motif previously reported.^10^, ^17^ The results showed the CXXCXXCX ac^4^C motif was conservative among hESCs, NEP and ME cells (Figure 1E). Moreover, consistent with the results of previous studies,^18^ the largest fraction of ac^4^C sites was within the coding sequence area (45%–49%), while the 5′‐UTR (12%–13%) and 3′‐UTR (2%–4%) regions of mRNAs were also enriched (Figure 1F). The topology of ac^4^C modification in hESCs was also preserved compared with ectodermal NEP cells (Figure 1G) or ME cells (Figure 1H). These results indicated that the data of acRIP‐seq was reliable.

In addition, we also found that mRNAs of core hESC pluripotency regulators, including *LIN28A*, *MYC*, *ZIC3* and *SOX2*, were modified with ac^4^C (Figure 1I). Lineage marker genes possessed ac^4^C modification, including *SOX1*, *NCAM1* and *CRABP1* in NEP cells (Figure 1J), and *TBXT* and *GSC* in ME cells (Figure 1K). These results indicated that the ac^4^C modification universally existed in human mRNAs. In hESCs, acRIP‐seq revealed a total of 31,998 peaks in 11,856 transcripts (an average of 2.70 peaks per transcript). Upon hESC differentiation towards NEP or ME cells, there were 45,803 peaks in 12,596 transcripts (an average of 3.64 peaks per transcript) in NEP cells and 30,905 peaks in 11,640 transcripts (an average of 2.65 peaks per transcript) in ME cells (Tables [Link], [Link], [Link]). When compared with hESCs, 40.52% of mRNAs in NEP cells contained three or more differential ac^4^C peaks. Meanwhile, only 15.93% of mRNAs in ME cells contained three or more differential ac^4^C peaks (Figure 1L). Furthermore, 25,730 peaks appeared in NEP cells, while 12,880 peaks appeared in ME cells compared with hESCs (Figure 1M). These results demonstrated that ac^4^C modification was dynamic between hESCs and hESC‐derived different germ layer cells and ac^4^C modification might exert a vital role during the differentiation of hESC to ectodermal cells.

### 3.2 ac^4^C modification enhances TE of mRNAs during ectodermal differentiation

The distribution of ac^4^C modification within the coding areas of mRNAs prompted us to speculate that mRNA acetylation may affect protein translation. We used Ribo‐seq to detect the mRNAs undergoing translation in hESCs and hESC‐derived different germ layer cells. The results of the principal component analysis showed that hESC‐derived NEP cells and ME cells were separate from hESCs (Figure S3A). Repetitive scatter plot analysis showed that the Pearson correlation of cells within the hESCs, hESC‐ME and hESC‐NEP group was greater than 0.9, indicating good reproducibility of the samples within each group (Figure S3B–D). In addition, ribosome footprints (RFs) were categorised into four categories: coding region, 5′‐UTR, 3′‐UTR and intron based on the alignment location of RFs on the coding gene. Statistical analysis showed that large numbers of RFs were distributed in the coding area (Figure S3E). The depth distribution of RFs in coding areas presented an obvious “high‐low‐low” three‐nucleotide periodic characteristic (Figure S3F–H). Ribo‐seq reads exhibited the expected codon distribution of RFs (Figure S3I–K). These results showed the Ribo‐seq data to be accurate and reliable.

**FIGURE 2:**
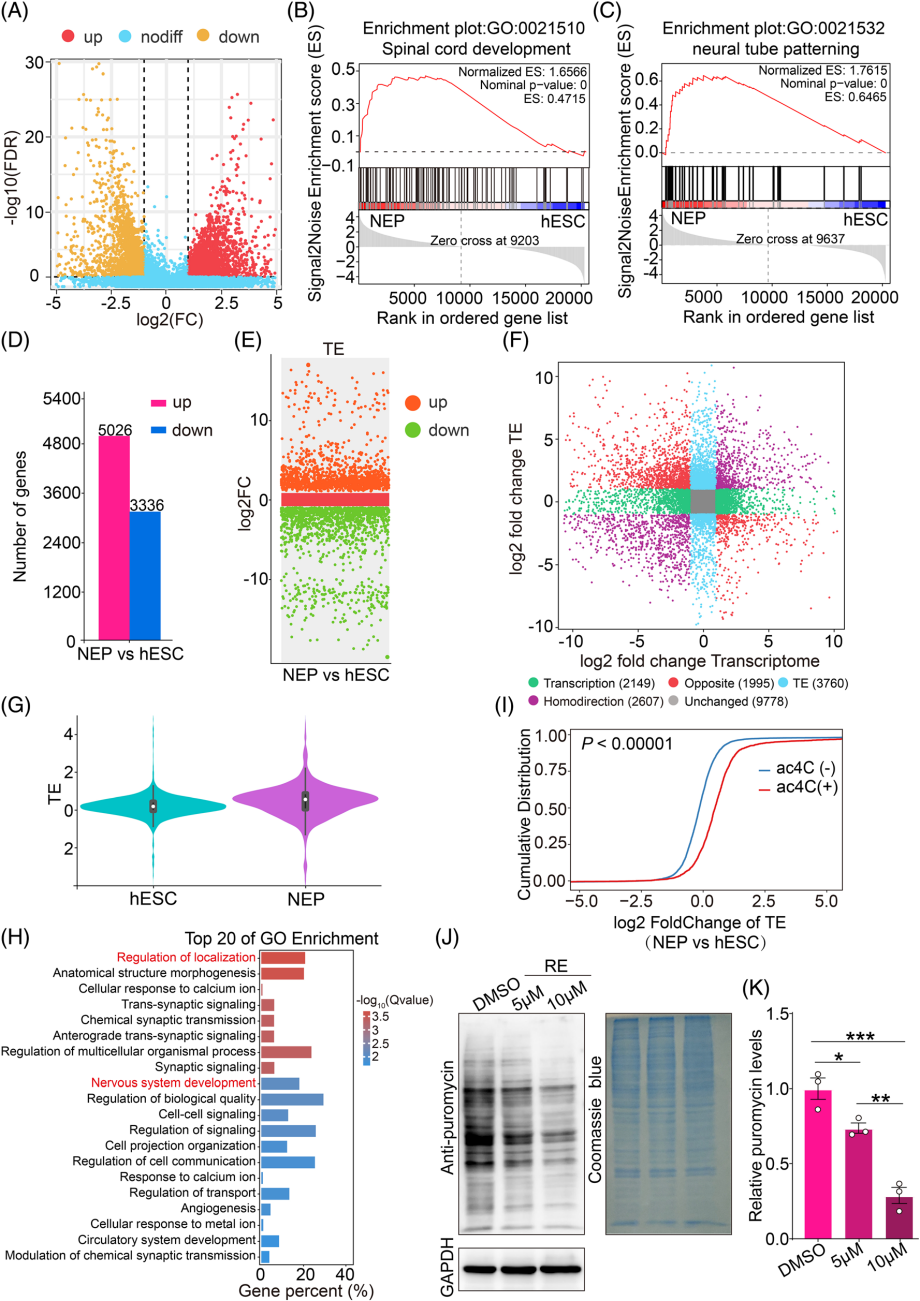
ac^4^C modification contributes to improve the translation efficiency of mRNAs in hESC‐derived NEP cells. (A) Volcano plot analysis of differential translation genes between NEP cells and hESCs. (B, C) GSEA profile of spinal cord development (B) and neural tube patterning (C) genes in hESCs and hESC‐derived NEP cells. (D) Statistical analysis of genes with translation efficiency fold change greater than 2 between hESCs and hESC‐derived NEP. (E) Differential scatter plots showed the upregulated (jacinth) and downregulated (green) genes in translation efficiency between hESCs and NEP cells. (F) Difference direction of translation efficiency (TE) and transcription level comparison and classification between NEP cells and hESCs. (G) Statistical analysis of the overall translation efficiency of genes between hESCs and NEP cells. (H) Gene ontology analysis of translation efficiency of genes in NEP cells versus hESCs. (I) Translation efficiency is plotted as cumulative fractions for mRNAs with (ac^4^C^+^) or without (ac^4^C^−^) ac^4^C modification in genes. Wilcoxon test (Mann–Whitney *U* test) in IBM SPSS Statistics 26 software was used to obtain *p* value. (J) Global translation of NEP cells with DMSO or different doses of RE (5 and 10 μM). Coomassie blue and GAPDH were used as control. (K) Quantitation of relative puromycin levels in (J) (*n* = 3).

According to the above study, the ac^4^C writer protein NAT10 and ac^4^C modification levels were obviously increased upon ectoderm differentiation, indicating that differentiation of ectoderm, but not ME, is associated with increased ac^4^C modification. To reveal the role of ac^4^C modification in the regulation of translation during ectodermal differentiation, we first performed the volcano map analysis of the genes with differences in translation level (FDR < 0.05, |log_2_FC| > 1) (Figure 2A) and then GSEA for the Ribo‐seq data for hESCs and NEP cells. The results showed that spinal cord development and neural tube patterning were enriched during ectodermal differentiation (Figure 2B,C). Previous studies showed that the ac^4^C modification could regulate the TE.^25^ Subsequently, we calculated the TE according to the formula previously reported.^26^ The results suggested that when hESCs differentiated into NEP cells, we identified a total of 8362 genes, 5026 genes of which increased the TE fold change and 3336 genes showed downregulate (|log_2_FC| > 1) (Figure 2D,E). Plotting TE against RNA‐seq data for hESCs and NEP cells, showed 2149 genes to be different at the transcript level only and 3760 genes to be different at translational efficiency only (Figure 2F). Moreover, when compared with hESCs, the overall TE of genes in NEP cells was higher (|log_2_FC| > 1) (Figure 2G). Gene ontology (GO) revealed that genes with TE fold change greater than 2 were mostly related to nervous system development and regulation of localization (Figure 2H). Next, we considered whether the enhancement of TE during the differentiation of hESCs into NEP cells was regulated by ac^4^C modification. A combined analysis with TE and genes of upregulated or those without ac^4^C acetylation in hESCs and NEP cells was performed. The results showed that the TE of ac^4^C^+^ mRNAs was obviously increased than that of ac^4^C^−^ mRNAs (Figure 2I). In addition, the level of TE directly affects the amount of nascent protein. If the ac^4^C modification in NEP cells directly affects mRNA translation, then lowering the level of this modification would decrease the amount of nascent protein in NEP cells. To verify this possibility, we treated NEP cells with RE, a specialised inhibitor of NAT10, and found that the amount of nascent protein in NEP cells was significantly reduced when ac^4^C modification was lowered and that the amount of nascent protein showed a concentration‐dependent decrease as the concentration of RE increased (Figures 2J,K and S4A–C). Together, these results showed that RNA TE was increased during ectodermal differentiation, and this increase was regulated by ac^4^C modification in NEP cells.

### 3.3 ac^4^C acetylation of NR2F1 catalysed by NAT10 contributes to promote ectoderm differentiation of hESCs

**FIGURE 3:**
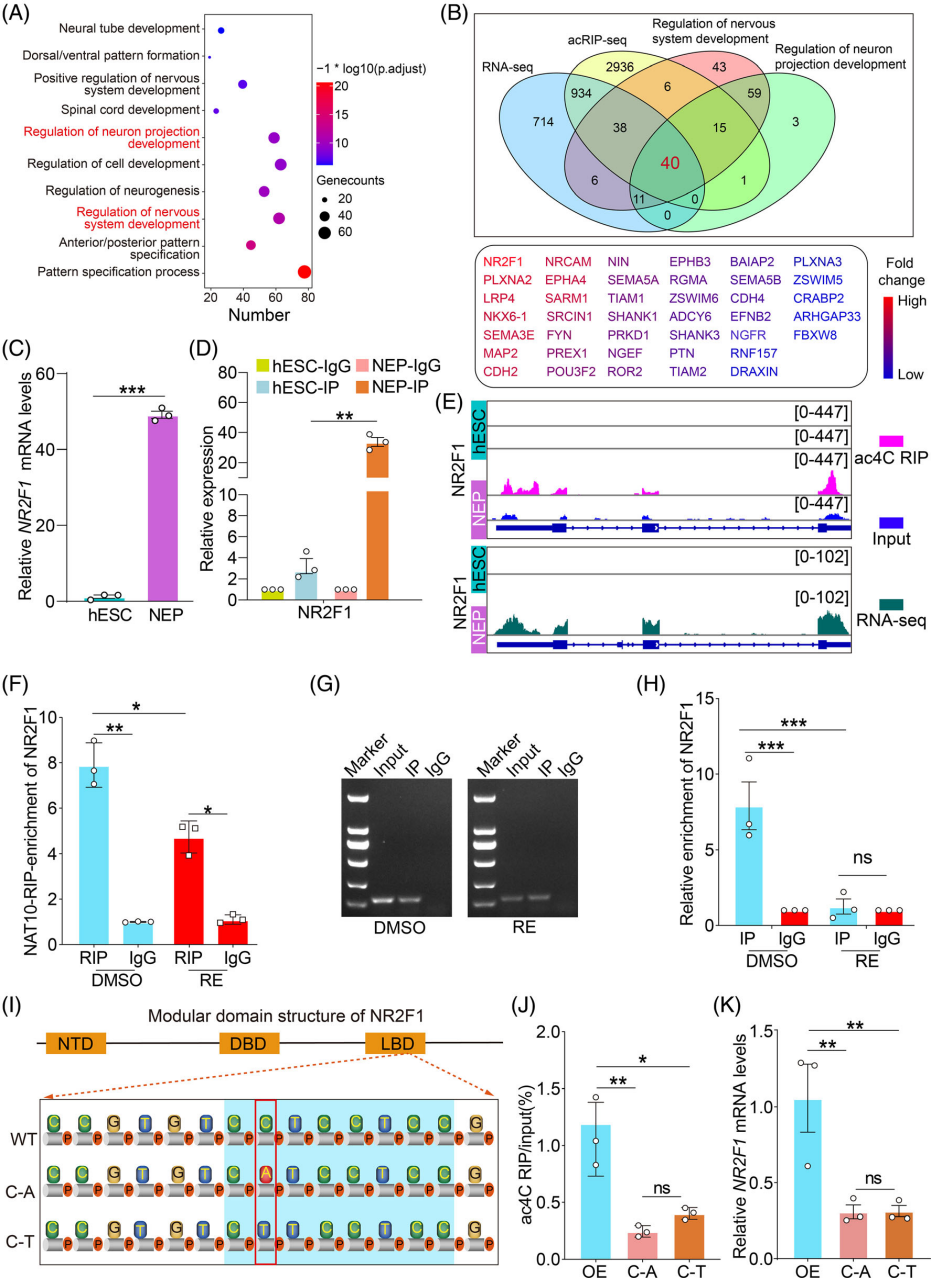
NAT10‐mediated ac^4^C modification promotes the expression of NR2F1 in NEP cells. (A) GO analysis of the upregulated genes from acRIP‐seq and RNA‐seq data in NEP cells and hESCs. (B) Ribo‐seq genes involved in the regulation of nervous system development and neuron projection development were overlapped by data from acRIP‐seq and RNA‐seq analyses (up). Heatmap analysis shows the fold change of 40 overlapping genes (down). (C) qRT‐PCR was used to verify the expression of NR2F1 in hESCs and hESC‐derived NEP cells. (D) acRIP‐qRT‐PCR was performed to test the ac^4^C modification levels of NR2F1 in hESCs and hESC‐derived NEP cells. (E) Peaks of NR2F1 ac^4^C and mRNA in hESCs and hESC‐derived NEP cells. (F) NAT10 immunoprecipitation followed by qRT‐PCR (NAT10 RIP‐qRT‐PCR) demonstrated that *NR2F1* mRNA interacted with NAT10. (G) RT‐PCR was used to test the product *NR2F1* mRNA of NAT10‐RIP. (H) acRIP‐qPCR was used to test the ac^4^C modification levels in the DMSO group and RE group. (I) Schematic representation of the positions of ac^4^C motifs within NR2F1. (J) qRT‐PCR analysis of changes in ac^4^C‐modified NR2F1 levels between wild‐type and mutant NR2F1. (K) Levels of NR2F1 transcripts in HEK293T cells transfected with NR2F1‐overexpressing plasmids or mutants. OE: NR2F1‐overexpressing cells; C‐A: mutant with a C‐A transition mutation; C‐T: mutant with a C‐T transition mutation. Error bars indicate mean ± SEM.

To explore the mechanism and regulation role of ac^4^C modification in ectodermal differentiation, we identified potential genes of the ac^4^C writer, NAT10, in NEP cells. We co‐analysed the acRIP‐seq and RNA‐seq data of NEP cells and found that genes with upregulated ac^4^C modification and transcript level were mainly involved in the regulation of nervous system development and neuron projection development (Figure 3A). The genes highlighted by acRIP‐seq, RNA‐seq and Ribo‐seq that were involved in nervous system development and neuron projection development included 40 genes with differential ac^4^C modification and high levels of translation (Figure 3B). Among these 40 genes, NR2F1 had the highest fold change of ac^4^C modification between NEP cells and hESCs (Figure 3B). qRT‐PCR and acRIP‐qRT‐PCR were used to further validate *NR2F1* expression (Figure 3C) and ac^4^C modification levels (Figure 3D), respectively, in hESCs and NEP cells. Integrative Genomics Viewer analysis demonstrated that *NR2F1* had obvious acetylation peaks in NEP cells, which were absent in hESCs (Figure 3E). To validate the interaction between NAT10 and *NR2F1* mRNA in NEP cells, we next carried out NAT10 RIP‐qRT‐PCR and RT‐PCR experiments in NEP cells. The results showed that *NR2F1* mRNA could be enriched in NEP cells by using the NAT10 antibody. At the same time, we used the RE, to start treatment on the fifth day of hESCs differentiation into NEP cells. After 2 days, we collected the treated cells and carried out the RIP test of NAT10. The results revealed that the level of *NR2F1* mRNA enriched by NAT10 was significantly downregulated (Figure 3F,G). Furthermore, to further investigate the effect of RE on the ac^4^C modification level of NR2F1, we used acRIP‐qRT‐PCR to detect the changes in the ac^4^C modification of NR2F1. The results showed a significant enrichment of *NR2F1* mRNA in ectodermal NEP cells. However, after treating NEP cells with specific inhibitors of NAT10, RE, for 2 days, the ac^4^C modification level of *NR2F1* mRNA obviously decreased (Figure 3H). Based on these results, we demonstrated an interaction between NAT10 and *NR2F1* mRNA in NEP cells. To further verify whether ac^4^C modification can regulate NR2F1 expression, we created NR2F1 point mutation vectors where the cytosine residues within the ac^4^C motifs were converted to either adenine (C‐A mutation) or thymine (C‐T mutation) (Figure 3I). HEK293T cells were transfected with equal amounts of NR2F1‐overexpressing (control) or mutant plasmids. The mutations caused drastically decrease of the ac^4^C levels (Figure 3J) and expression levels (Figure 3K) of NR2F1, confirming that ac^4^C modification contributed to the increase in NR2F1 expression. Together, these results indicate that ac^4^C acetylation of NR2F1 catalysed by NAT10 promotes ectoderm differentiation of hESCs.

### 3.4 NAT10 acetylates *NR2F1* mRNA to enhance its stability and translation during ectodermal differentiation

**FIGURE 4:**
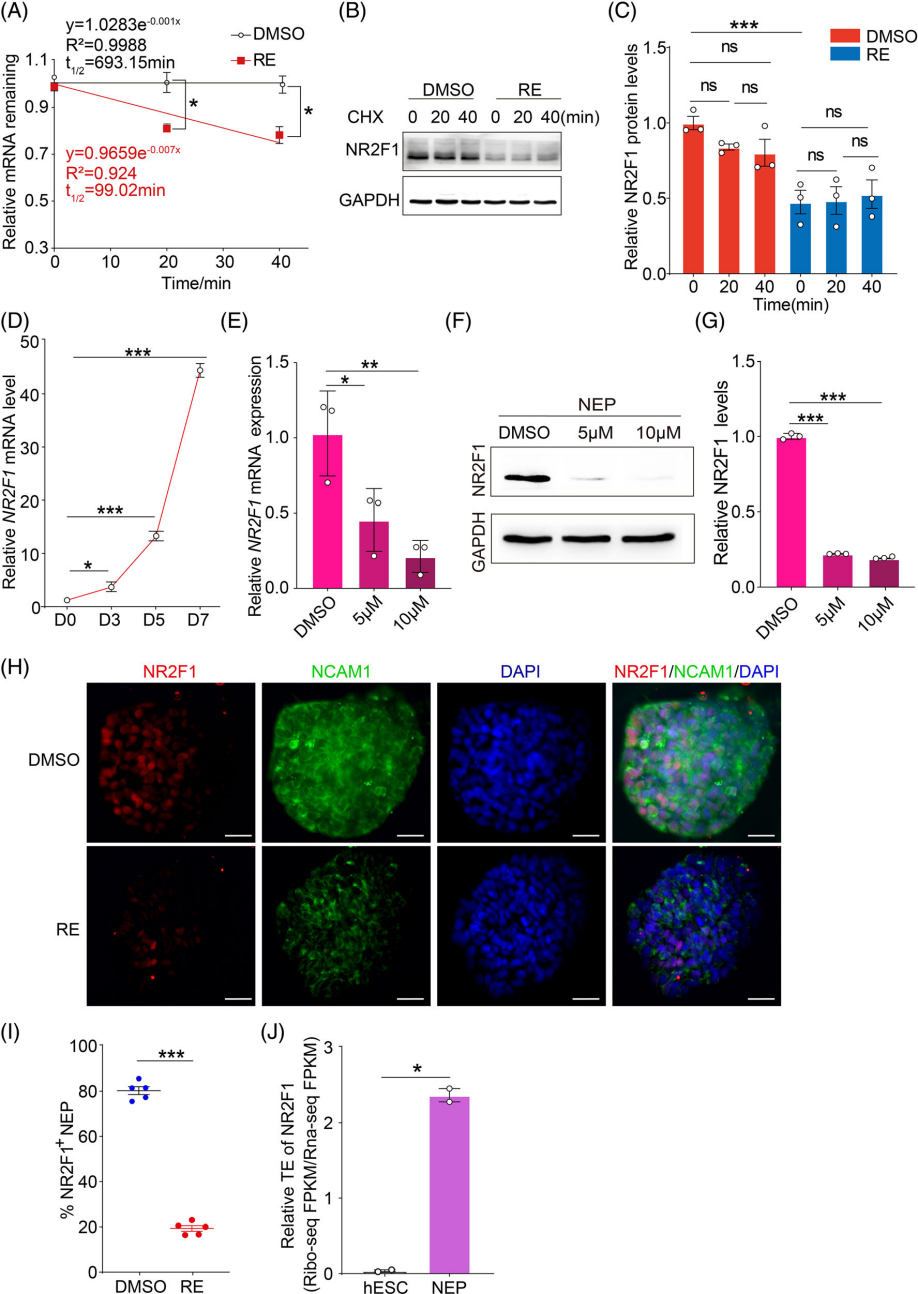
Acetylation of *NR2F1* mRNA increases its stability during translation in ectodermal differentiation. (A) Reducing *NR2F1* mRNA half‐life by inhibiting NAT10 in hESC‐derived NEP cells. RE: remodelin hydrobromide. (B) The protein levels of NR2F1 with the treatment of CHX (100 μg/mL) in NEP cells with or without RE. (C) Quantification of relative NR2F1 levels in (B) using Image J software. (D) qRT‐PCR determined *NR2F1* mRNA levels in hESCs and hESC‐derived NEP cells at different times (Days 0, 3, 5 and 7). (E, F) The expression levels of NR2F1 in NEP cells treated with DMSO or different doses of remodelin hydrobromide were tested by qRT‐PCR (E) and Western blotting (F). (G) Quantification of the relative expression levels of NR2F1 in (F). (H) Immunofluorescence assay was performed on DMSO‐ or remodelin hydrobromide‐treated NEP cells on Day 7 using an anti‐NR2F1 antibody (red) and anti‐NCAM1 antibody (green). DAPI (blue) was used to label the nucleus. RE: remodelin hydrobromide. Scale bar = 50 μm. (I) Quantification of NR2F1‐positive DMSO‐ or remodelin hydrobromide‐treated NEP cells at Day 7. RE: remodelin hydrobromide. (J) The translation efficiency of NR2F1 was calculated by Ribo‐seq analysis and RNA‐seq analysis. *n* = 2. Error bars display mean ± SEM.

Having shown that ac^4^C affects the post‐transcription of mRNAs, we next studied the mechanisms that limit the abundance of mature mRNA. We first explored changes in mRNA stability in RE (a NAT10‐specific inhibitor)‐treated NEP cells. RNA decay assays indicated that relatively fewer NR2F1 transcripts remain in RE‐treated cells compared with DMSO‐treated cells and its half‐life was reduced from 693.15 to 99.02 min (Figure 4A). Previous studies have shown that NAT10 not only possesses RNA acetyltransferase activity, but also regulates the expression of histones.^27^, ^28^, ^29^ To verify whether NAT10 can also regulate the stability of NR2F1 protein, we used the protein synthesis inhibitor CHX for protein stability testing, and the results showed that NR2F1 protein showed no significant changes in both the control and the NAT10 specific inhibitor group (Figure 4B,C). The foregoing studies revealed that NAT10 could not affect NR2F1 protein stability but could increase *NR2F1* mRNA stability through ac^4^C modification. In addition, we found that the mRNA (Figure 4D) and protein levels (Figure 6E) of NR2F1 gradually increased from Day 3 to Day 7, indicating that the level of NR2F1 translation increased cumulatively during NEP cell differentiation. When NEP cells were treated with RE, the mRNA (Figure 4E) and protein levels (Figure 4F,G) of NR2F1 declined significantly. Immunofluorescence staining showed that RE obviously reduced the percentage of NR2F1^+^NEP cells on Day 7 (Figure 4H,I). In addition, the ratio of Ribo‐seq FPKM/RNA‐seq FPKM increased more in NEP cells than in hESCs, indicating that the TE of NR2F1 was significantly higher during ectodermal differentiation of hESCs (Figure 4J). The above data suggested that NAT10 acetylated *NR2F1* mRNA promotes stability and translation during the differentiation of hESCs to NEP cells.

### 3.5 NR2F1 promotes ectodermal differentiation of hESCs by accelerating nuclear export of YAP1

**FIGURE 5:**
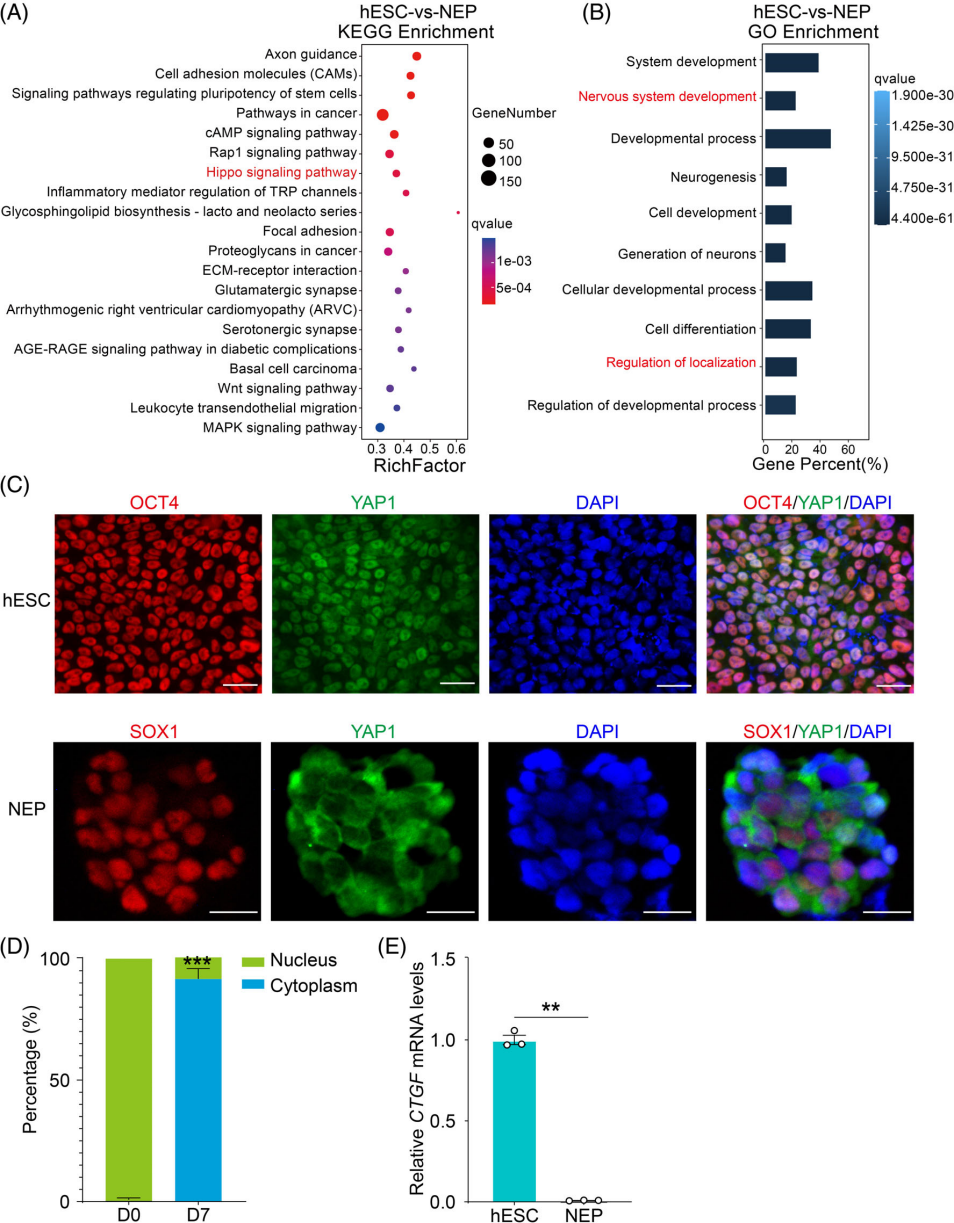
The Hippo pathway is activated during ectodermal differentiation. (A) The Hippo pathway was highly enriched during the differentiation of hESCs to NEP cells according to KEGG analysis of the RNA‐seq data of NEP vs hESCs cells. (B) Gene ontology analysis of the differential genes in the RNA‐seq data of NEP cells and hESCs. (C) Localization of YAP1 in hESCs and Day 7 NEP cells. OCT4 was a marker of hESCs and SOX1 was a marker of NEP cells. Scale bar = 50 μm. (D) Statistical analysis of the cytoplasmic and nuclear localization of YAP1 in hESCs or Day 7 NEP cells. (E) CTGF expression in hESCs and hESC‐derived NEP cells was analysed by qRT‐PCR. Error bars display mean ± SEM.

KEGG analysis of RNA‐seq data of NEP cells versus hESCs indicated that the Hippo pathway was enriched (Figure 5A). GO analysis of the genes with differential expression levels between NEP cells and hESCs showed that nervous system development and regulation of cellular localisation were highly enriched (Figure 5B). These results demonstrated that the Hippo pathway might exert a significant role in regulating neural development as well as cellular localization. Nuclear export of YAP1 can stimulate the Hippo pathway activation.^30^ To further elucidate the role of the YAP1 during the differentiation of hESCs to NEP cells, we first investigated changes in the localization of YAP1. Immunofluorescence assay revealed that YAP1 was located in the nucleus of hESCs, while its localization was mostly on the cytoplasm in NEP cells at day 7 (Figure 5C,D). In addition, the mRNA level of the YAP1‐TEAD downstream target gene, connective‐tissue growth factor (*CTGF*), was clearly lower in NEP cells than in hESCs, which indicated the YAP1 was inactivated (Figure 5E). These results demonstrated activation of the Hippo pathway during hESC differentiation to NEP cells.

**FIGURE 6:**
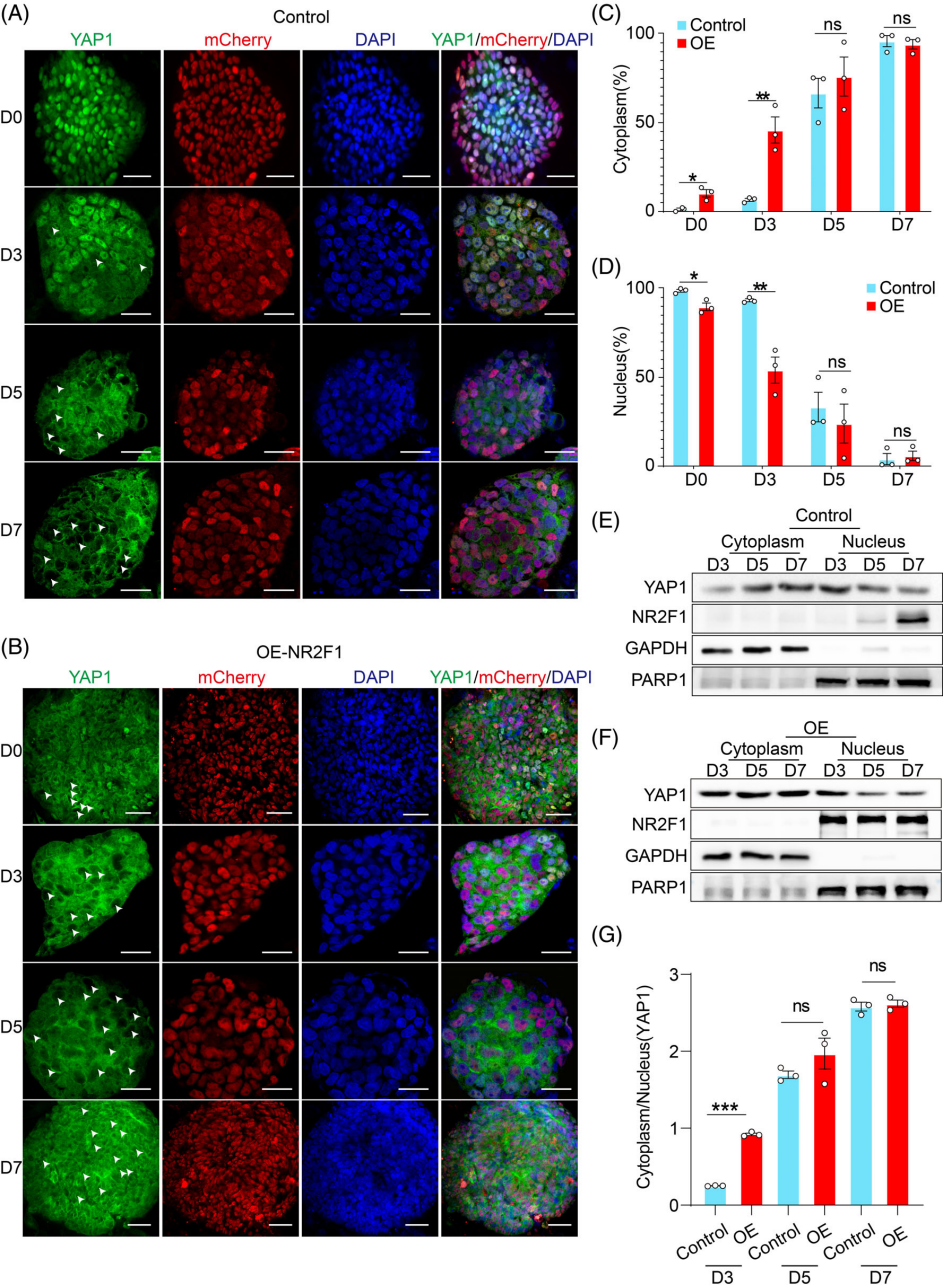
NR2F1 accelerates the nuclear export of YAP1 during the differentiation of hESCs into NEP cells. (A, B) Localization of YAP1 during the differentiation of hESCs (A) or OE‐NR2F1 hESCs (B) into NEP cells was analysed by immunofluorescence staining at different times (Days 0, 3, 5 and 7). Scale bar = 50 μm. (C, D) Statistical analysis of YAP1 localization in the cytoplasm (C) and nucleus (D) at different times (Days 0, 3, 5 and 7) during differentiation of control hESCs or OE‐NR2F1 hESCs into NEP cells. (E, F) Western blot analysis of changes to nuclear and cytoplasmic localization of YAP1 and NR2F1 at different times (Days 3, 5 and 7) during hESC (E) or OE‐NR2F1 hESC (F) differentiation into NEP cells. (G) The ratio of cytoplasmic to nuclear YAP1 was statistically analysed in (E) and (F). Error bars display mean ± SEM.

To determine the link of NR2F1 and the Hippo pathway on hESC self‐renewal, we constructed NR2F1 overexpression hESC lines (OE‐NR2F1 hESCs) (Figure S5A). The overexpression levels of NR2F1 were validated by qRT‐PCR (Figure S5B) and western blotting (Figure S5C). We found that when NR2F1 was overexpressed, nucleus‐to‐cytoplasm translocation of YAP1 was observed in a small number of hESCs (10.26%) at Day 0 (Figure 6B). Moreover, the expression level of key factor OCT4 for self‐renewal was downregulated, but there was no significant difference compared to the control group (Figure S5D). Besides, although the number of colonies in the NR2F1 overexpression group did not significantly differ from the control group, the cells in the NR2F1 overexpression group were not compact enough (Figure S5A,E). The above studies suggested that NR2F1 might not be necessary for maintaining the self‐renewal of hESCs.

**FIGURE 7:**
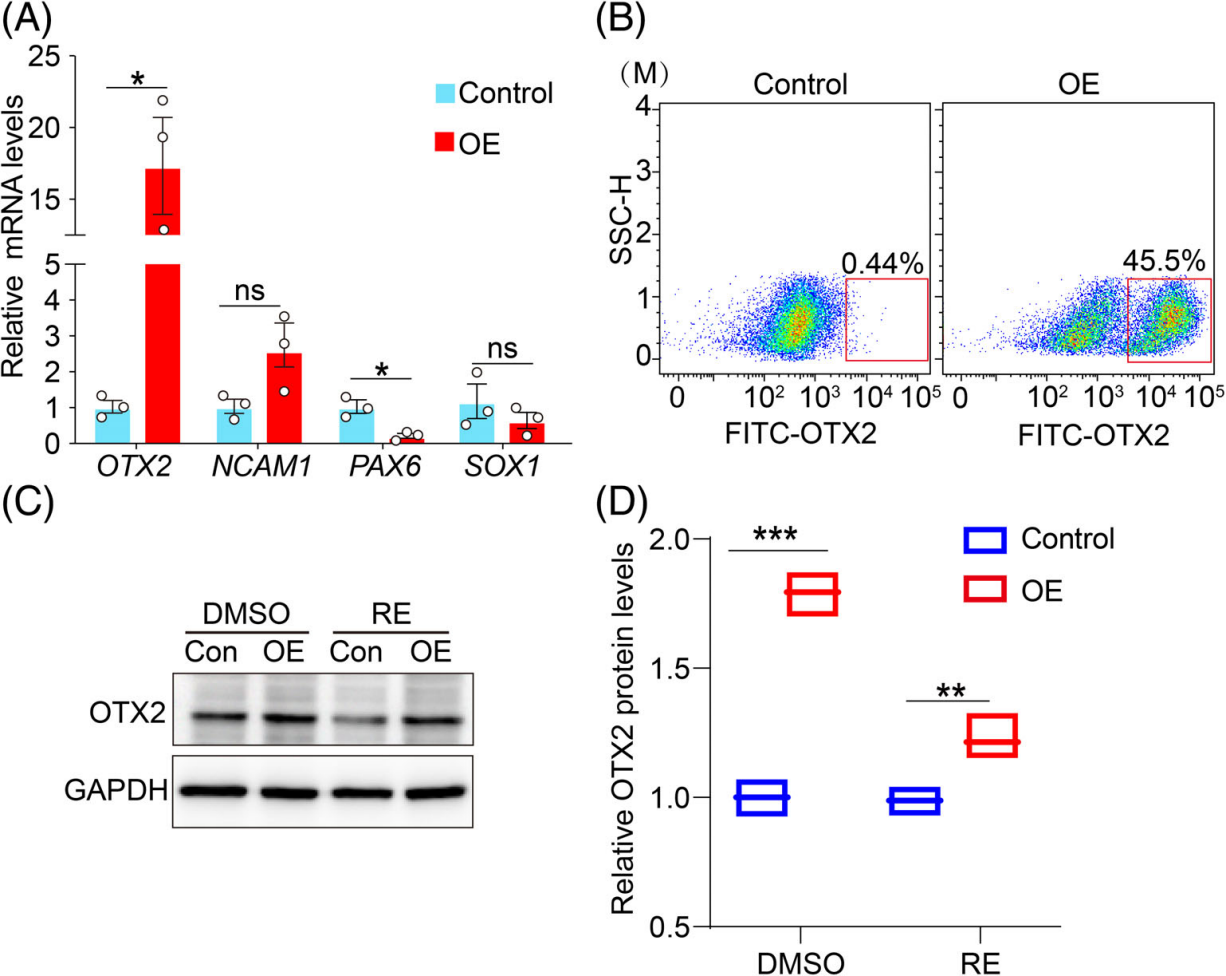
NR2F1 promoted the ectodermal differentiation of hESCs. (A) The expression levels of ectodermal NEP cell markers (OTX2, NCAM1, PAX6 and SOX1) were detected by qRT‐PCR in control and OE cells. Control: OE‐control hESC‐derived NEP cells. OE: OE‐NR2F1 hESC‐derived NEP cells. (B) FACS plots depicting FITC‐OTX2 fluorescence in control and OE cells. Control: OE‐control hESC‐derived NEP cells. OE: OE‐NR2F1 hESC‐derived NEP cells. (C) Overexpression of NR2F1 could partially compensate for the decrease in RE‐induced ectoderm differentiation capacity. RE: remodelin hydrobromide. (D) Quantification of relative OTX2 levels in (C).

Previous studies showed that NR2F1 point mutation caused neurodevelopmental disorders diseases such as Bosch‐Boonstra‐Schaaf optic atrophy syndrome (BBSOAS) in vivo,^31^ which suggests that the NR2F1 was vital in the development of the nervous system. However, the in vitro role of NR2F1 during hESCs differentiation into NEP cells was unclear. To clarify the link between NR2F1 and the Hippo pathway in hESCs differentiation into NEP cells, we performed ectodermal differentiation on both the control hESCs and OE‐hESCs. The results showed that the control group exhibited neural tube‐like structures, which were further increased in the NR2F1 overexpression group. During ectodermal differentiation of control hESCs, a small amount of YAP1(6.58%) translocated from the nucleus to the cytoplasm on the third day of differentiation and as ectodermal differentiation progressed, this translocation gradually increased (Figure 6A). When NR2F1 was overexpressed, nucleus‐to‐cytoplasm translocation of YAP1 was obviously increased on Day 3 compared to the control group (Figure 6C,D). These results were corroborated by cytoplasmic and nuclear extraction assays during ectodermal differentiation (Figure 6E–G). The above results indicate an interaction between NR2F1 and YAP1. Moreover, qRT‐PCR analysis showed that overexpression of NR2F1 markedly elevated the mRNA level of the early neural marker, *OTX2* (Figure 7A). Flow cytometry analysis further confirmed that overexpression of NR2F1 increased the percentage of OTX2^+^ cells from 0.44% to 45.5% (Figure 7B). Furthermore, the overexpression of NR2F1 partially rescued the decrease in RE‐induced ectoderm differentiation capacity (Figure 7C,D). Together, these results reveal that NR2F1 accelerates the nuclear export of YAP1, thereby promoting ectodermal differentiation.

## 4 DISCUSSION

ac^4^C modification of mRNA was first identified in 2018^7^ and since then, increasing numbers of studies have focused on this new type of RNA modification. ac^4^C modification has been found in numerous species and it regulates varied biological processes, such as oocyte maturation,^32^ spermatogenesis,^14^ cancer cell progression^11^ and osteogenic differentiation.^33^ Moreover, ac^4^C modification is also linked to human diseases, such as ischemic heart disease,^18^ bladder cancer^10^ and gastric cancer,^34^ and has potential as an effective therapeutic target. Previous research has largely concentrated on NAT10's acetyltransferase properties. For instance, NAT10 controls mitosis by acetylating Eg5 at K771^28^ and increases the transcription of human telomerase reverse transcriptase to enhance telomerase activity.^27^ In 2018, Arango et al. found that NAT10 was also involved in increasing mRNA ac^4^C modification in mammals.^7^ Remodelin, a specific inhibitor of NAT10, has been widely reported that it can significantly decrease the ac^4^C level.^25^, ^35^, ^36^ Our results also demonstrated that when we used the RE to treat the hESCs for 2 days, the ac^4^C modification levels were significantly reduction (Figure S6A,B). However, the roles of NAT10‐mediated ac^4^C modification in hESCs and hESC‐derived different germ layer cells have not been reported.

We applied acRIP‐seq technology to hESCs and hESC‐derived different germ layer cells, to generate the first large‐scale resource of ac^4^C events. We demonstrated that mRNA acetylation was widely distributed and dynamic during early differentiation of hESCs to germ layer cells. This dynamic change may be due to the presence of other ac^4^C recognition proteins or erasing proteins. This is because when hESCs or NEP cells were treated with remodelin, the level of ac^4^C modification decreased, but a small amount of this modification still remained (Figures S4A and S6A). In addition, it is generally known that the process of translation from mRNA to protein often involves post‐transcriptional modification, but this dynamic process cannot be accurately reflected by standard transcriptome analyses. The advent of Ribo‐seq technology provides an opportunity to study the translatome. Mistranslation of mRNA leads to neurodevelopmental diseases^37^ and neurodegenerative disorders.^38^ To further examine the function of ac^4^C modification and mRNA translation in regulating neural development, we co‐analysed acRIP‐seq, RNA‐seq and Ribo‐seq data about the regulation of nervous system development and neuron projection development. In addition, combined with RIP‐qRT‐PCR, NR2F1 was identified as an interaction factor with NAT10 in NEP cells. NR2F1 contains three conserved domains. The complex ligand‐binding domain (LBD) not only binds to ligands but also directly engages co‐regulator proteins.^39^ In this study, when the point mutation of the ac^4^C motif occurred in the LBD of NR2F1, its transcriptional activity and ac^4^C modification level were significantly reduced. And when the level of ac^4^C modification was reduced by using a NAT10‐specific inhibitor, the decreased expression of NR2F1 resulted in inhibited differentiation of ectodermal cells. This is consistent with previous reports that *NR2F1* mutations cause BBSOAS in vivo.^31^, ^40^ These observations indicate that NR2F1 exerts a crucial function in ectodermal cells. In addition, previous studies have confirmed that *NR2F1* expression is high in the dorsal and low in the ventral of the brain.^41^, ^42^ However, another study showed that overexpression of NR2F1 promotes ventral molecular properties in the murine brain cortex.^43^ Here, we showed that the expression of NR2F1 was beneficial to dorsal gene expression during ectodermal differentiation, while overexpression of NR2F1 obviously increased the level of OTX2, a marker of ventral forebrain and midbrain.^44^ Meanwhile, the mRNA level of the dorsal gene, *PAX6*, was decreased (Figure 7A). These results elucidated the dose‐dependent manner of NR2F1 regulation of ventral/dorsal patterning and the fine‐tuning mechanism that enables cells to differentiate to specific fates.

The KEGG analysis of RNA‐seq data from NEP cells versus hESCs showed that the Hippo pathway was enriched during the ectodermal differentiation of hESCs, indicating that the Hippo pathway was critical in ectodermal differentiation. The Hippo pathway is involved in regulating a variety of molecular signals such as cell polarity,^30^ mechanical force^45^ and biophysical state.^46^ When the Hippo pathway is ‘off’, YAP1 enters the nucleus and binds to the transcription factor, TEAD.^46^ Upstream kinases in the Hippo pathway, such as mammalian Ste‐20‐like kinase 1/2 (MST1/2), promote nuclear export of YAP1 and inhibit YAP1 transcriptional activation by phosphorylation.^47^, ^48^, ^49^ However, non‐receptor protein tyrosine phosphatase 14 (PTPN14) regulates this translocation independently of PTPN14 phosphorylation in MCF10A cells.^50^ Zaltsman et al. reported that YAP1 localization is directly controlled by angiomotin (AMOT) and is independent of YAP1 phosphorylation.^51^ Here, we found the nucleus to cytoplasm translocation of YAP1 gradually increased from Day 3 to Day 7 during the differentiation of ectodermal NEP cells and that overexpression of NR2F1 accelerated this effect. This was consistent with the results of the recently published article, which suggested that when overexpression the NR2F1, the recruitment of histone activating marks such as H3K27ac to the yap promoter was decreased, and the Yap‐tead complex was suppressed.^52^

In conclusion, we demonstrated that ac^4^C modification was dynamic and enriched in hESC‐derived ectodermal cells. Furthermore, the ac^4^C modification enhanced the TE of mRNAs during ectodermal differentiation. We also identified that *NR2F1* mRNA was interacted with NAT10 and that *NR2F1* mRNA was acetylated by NAT10 to enhance its stability and translation during ectodermal differentiation, which was beneficial for increasing NR2F1 protein expression. NR2F1 accelerated the nuclear export of YAP1, which promoted the ectodermal differentiation of hESCs. These findings offer a novel perspective into the mechanism of early hESC differentiation and assist in the treatment of ectodermal differentiation defects‐related diseases.

## AUTHOR CONTRIBUTIONS

**Junbang Ge**: Conceptualization; methodology; formal analysis; investigation; writing—original draft; writing—review and editing. **Zhaoxia Wang**: Supervision. **Ji Wu**: Conceptualization; supervision; writing—review and editing; project administration; funding acquisition.

## CONFLICT OF INTEREST STATEMENT

The authors declare no conflict of interest.

## Supporting information


**DATA S1.** Supplementary Information.

**FIGURE S1.** Identification of hESC‐derived NEP cells and ME cells. (A) A scheme for the differentiation of hESCs to ME cells and NEP cells. S: SB431542; C: CHIR99021; D: DMH1; Representative photos at the indicated stages are shown. Scale bar = 50 μm. (B) RT‐PCR analysis of SOX1, HOXA3, NCAM1 in NEP cells and TBXT and MIXL1 expression in ME cells. GAPDH served as an endogenous normalizer. (C) qRT‐PCR analysis of *OCT4*, *SOX1*, *HOXA3* and *NCAM1* mRNA levels in NEP cells at day 7 and hESCs (normalised to GAPDH). (D‐F) qRT‐PCR analysis of *OCT4* (D), *TBXT* (E) and *MIXL1* (F) at different times (0 h, 12 h, 24 h and 36 h) during the differentiation of hESCs into ME cells. (G) Immunofluorescence assay was carried out using the anti‐OCT4 antibody (red), the anti‐HOXA3 antibody (green) and the anti‐TBXT antibody (green) on hESCs, the anti‐HOXA3 antibody (green), the anti‐OCT4 antibody (red) on NEP cells and anti‐TBXT antibody (green), the anti‐OCT4 antibody (red) on ME cells. DAPI was used to label the nuclei (blue). Scale bar = 50 μm. Data display the mean ± SEM.

**FIGURE S2.** Gating strategy for cell purification. (A–C) After gating by SSC and FSC, DAPI was used to exclude dead cells. Then CD29 was used to exclude mouse feeder cells. The remaining live hESCs (A), ME cells (B) and NEP cells (C) were retrieved for further analysis. SSC: side scatter. FSC: forward scatter.

**FIGURE S3.** Quality control of Ribo‐seq data. (A) Three‐dimensional PCA plot of hESCs and hESC‐derived NEP cells and ME cells. (B–D) Repetitive scatter plots were used to evaluate the repeatability of hESCs (B), ME cells (C) and NEP cells (D) within the group. (E) Distribution of ribosome footprints (RFs) in coding genes. (F–H) Distribution of the abundance of all ribosome footprints (RFs) in hESCs (F), ME (G) and NEP cells (H) around the start and end codons. (I–K) Bar plots displaying the distribution of codons for RFs of each length in hESCs (I), ME (J) and NEP cells (K).

**FIGURE S4.** The decrease in ac^4^C modification levels leaded to a decrease in the synthesis of new proteins. (A) mRNA dot blot analysis the expression levels of ac^4^C modification in hESCs, NEP cells and RE‐treated NEP cells. (B) Global translation of DMSO‐treated NEP cells and RE‐treated NEP cells (5 μM). Coomassie blue and GAPDH were used as control. RE: remodelin hydrobromide. (C) Quantitation of relative puromycin levels.

**FIGURE S5.** NR2F1 is not the necessary factor for self‐renewal of hESCs. (A) Bright field pictures of OE‐control hESCs and OE‐NR2F1 hESCs. (B, C) NR2F1 mRNA (B) and protein (C) levels in control and OE‐NR2F1 human ESCs. Error bars display mean ± SEM. (D) The expression levels of *OCT4* mRNA were tested used the qRT‐PCR method. (E) The number of clones was statistically analysed in control group and OE‐NR2F1 group.

**FIGURE S6.** Remodelin hydrobromide induced the decrease of ac^4^C modification. (A) mRNA dot blot analysis the ac^4^C modification levels in hESCs with different doses of RE for 2 days. RE: remodelin hydrobromide. (B) Quantitation of relative ac^4^C levels in (A) (n = 3).

**TABLE S1.** Supplementary Table.

**TABLE S2.** Supplementary Table.

**TABLE S3.** Supplementary Table.

**TABLE S4.** Supplementary Table.

## Data Availability

Raw sequence data of acRIP‐seq, Ribo‐seq and RNA‐seq have been submitted to the NCBI sequence read archive under accession number GSE225052.
