# Supplementary material for: NAT10‐mediated ac4C modification promotes ectoderm differentiation of human embryonic stem cells via acetylating NR2F1 mRNA
Source: Cell Prolif. 2023 Dec 2;57(4):e13577. doi: 10.1111/cpr.13577 (PMC10984107; doi:10.1111/cpr.13577)
Supplement: Supplementary file 1 — DATA S1. Supplementary Information. [file CPR-57-e13577-s007.docx]

**Supplemental materials and methods**

**2.1 Cell culture**

hESCs (Line H9, passages 39–55, Shanghai Chuan Qiu Biotechnology Co.,Ltd., China) were cultured on a feeder layer of mouse embryonic fibroblasts. Mitomycin C (10 μg/mL, 51854, Cell Signaling Technology) was applied to the mouse embryonic fibroblasts for 3 h. The hESC medium consisted of Dulbecco's Modified Eagle’s Medium/Nutrient Mixture F-12 (DMEM/F-12, 11330032, Gibco), 1% GlutaMax™ (35050061, Gibco), 0.1 mM β–mercaptoethanol (M3148, Sigma), Knockout serum replacement (KSR, 10828028, Gibco), 10 ng/mL basic fibroblast growth factor 2 (FGF2, 234-FSE, R&D Systems) and 1% Nonessential amino acid solution (NEAA, 11140050, Gibco).

HEK293T cells were cultured in Dulbecco's modified Eagle's medium (DMEM) (11965092, Gibco) supplemented with 1× penicillin/streptomycin (15070063, Gibco) and 10% fetal bovine serum (FBS).

**2.2 Plasmid construction and lentivirus infection**

To generate overexpression vector of NR2F1, a full-length NR2F1 cDNA was cloned into the overexpression vector using a recombinational cloning kit (HANBIO, HB-infusion). Mutations were generated using the method of primer amplification. In short, the target fragment was divided into fragment 1 and fragment 2 according to the mutation site. After obtaining fragment 1 and fragment 2, the target fragment was amplified by C-A primers or C-T primers (Table S1) and then cloned into the overexpression vector. DNA sequencing was used to verify the existence of mutations in the created plasmids. In accordance with the guidelines provided by the Lipo8000 kit (Beyotime, C0533), the plasmid was transfected into HEK293T cells. OBiO Technology Co., Ltd. (Shanghai, China) performed the lentivirus packaging assay. When hESCs were passaged, the cells were treated with lentivirus for 24 h. To obtain stable hESC lines, cells were cultured with puromycin (10 μg/mL, Beyotime Biotechnology, China) for 5 days.

**2.3 Flow cytometry**

For flow cytometric sorting of hESCs, ME and NEP cells, single cells were obtained using TrypLE™ Express and washed with FACS phosphate buffer (1× PBS +1% FBS+0.01% HEPES). Cells were stained with an anti-mouse/rat CD29-APC antibody (1:200, Biolegend) for 30 minutes at 4°C and then washed with FACS phosphate buffer. Dead cells were removed using DAPI (0.5–1 μg/mL) staining. The target cells were collected using a FACS Aria III (BD Bioscience) with 652-nm and 405-nm excitation lasers. Flow cytometry data analysis using FlowJo software (v10.8.1).

The percentage of OTX2^+^cells in control hESC-derived NEP cells or OE-NR2F1 hESC-derived NEP cells was determined by flow cytometric analysis using the Transcription Factor Staining kit (00552300, Invitrogen, USA). In brief, TrypLE™ Express was used to separate cells into single cells and fixed in fixing/breaking working fluid at 4°C overnight. Cells were then treated with a human OTX2 Alexa Fluor® 488-conjugated antibody (1:200, R&D System) at 25°C for 3 h. Stained cells were analyzed using a FACS Cytoflex S (Beckman CoutlerCo., Ltd).

**2.4 Immunofluorescence assay**

The cells were treated with 4% paraformaldehyde for 30 min, rinsed with PBS, and after blocking in 3% BSA with 0.2% Triton X-100 for 1 h at 25°C, cells were exposed to primary antibodies for overnight at 4°C. Primary antibodies included goat anti-SOX1 (1:500, R&D Systems), mouse anti-TBXT (1:100, Santa Cruz Biotechnology), mouse anti-YAP1 (1:200, Santa Cruz Biotechnology), rabbit anti-NR2F1 (1:200, Abcam), rabbit anti-OCT4 (1:200, Santa Cruz Biotechnology), mouse anti-OCT4 (1:200, Cell Signaling Technology), mouse anti-NCAM1 (1:200, Santa Cruz Biotechnology) and rabbit anti-HOXA3 (1:500, Sigma-Aldrich). DAPI (10 μg/mL, Sigma) was used to label the nuclei after 1.5 h of appropriate fluorescently labeled secondary antibody application. Leica DMI3000 B and Nikon Ni-E A1 HD25 were used to obtain the images.

**2.5 Reverse transcription-polymerase chain reaction (RT-PCR) and qRT-PCR**

Total RNA was extracted using a TRIzol reagent. cDNA was synthesized using HifairIII 1^st^ strand cDNA Synthesis SuperMix (Yeasen, Co., Ltd.). qRT-PCR was performed using the Applied Biosystems 7500 instrument. The primers were described in Table S1.

**2.6 Western blotting**

The western blotting assay was performed according to the previous reports.^1, 2^ Briefly, RIPA lysis buffer from Yeasen Co., Ltd. was used to lyse the cells. The identical quantities of proteins were put onto 12% SDS-PAGE, electrophoresed, and then transferred to PVDF. The membranes were first washed once with Tris-buffered saline (TBS; 1 M Tris-HCl, 0.15 M NaCl), and then blocked for 2 hours at 25°C with 5% non-fat milk in TBS containing 0.05% Tween-20 (TBST). The membranes were then treated overnight at 4°C with the primary antibodies listed below: rabbit anti-NAT10 (1:3000, Proteintech), mouse anti-YAP1 (1:3000, Santa Cruz Biotechnology), rabbit anti-NR2F1 (1:3000, Abcam), mouse anti-OTX2 (1:4000, Santa Cruz Biotechnology), mouse anti-GAPDH (1:10,000, Proteintech), rabbit anti-puromycin antibody (1:2000, Abclonal) and mouse anti-β-actin (1:5000, Proteintech). The blots were washed thrice in TBST before being exposed to an enhanced chemiluminescence reagent after being incubated with the appropriate horseradish peroxidase-conjugated second antibodies for 1.5 hours at 25°C. Using a FUSION FX imager from Vilber (France), the membranes were scanned and captured. As internal controls for protein analysis, GAPDH or β-actin were utilized.

**2.7 Separation of cytoplasmic and nuclear protein extracts**

A NE-PER Nuclear and Cytoplasmic Extraction kit (Thermo Scientific, USA) was used to accomplish a step-by-step separation of the cytoplasmic and nuclear protein extracts. As a nuclear control, a PARP1 antibody (1:2000, Santa Cruz Biotechnology) was applied. As a cytoplasmic control, a GAPDH antibody (1:10,000, Proteintech) was employed.

**2.8 acRIP-seq data analysis**

The CloudSeq Inc. (Shanghai, China) completed the analysis of the acRIP-seq data. Briefly, Illumina NovaSeq 6000 sequencing was performed to harvest paired-end reads and Q30 was used for quality control. After trimming 3′ adaptors and removing low-quality reads using cutadapt (v1.9.3), Hisat2 (v2.0.4)^3^ was used to align the clean reads to the HG19. MACS^4^ was used to identify the acetylated sites on mRNAs. DiffReps^5^ was used to identify the differentially acetylated sites (*P*<0.00001, |Log_2_fold change|>1). GO analysis was performed on the differentially acetylated protein-coding genes.

**2.9 RNA-seq**

Gene Denovo Biotechnology Co. (Guangzhou, China) completed the RNA-seq assay. In short, total RNA was extracted using TRIzol. The RNA quality was evaluated using an Agilent 2100 Bioanalyzer (Agilent Technologies, Palo Alto, CA, USA). To enrich mRNA, oligo (dT) beads were utilized. The NEBNext Ultra RNA Library Prep Kit from New England Biolabs was used to create the libraries. Paired-end deep sequencing was carried out on the Illumina NovaSeq6000 platform (Illumina, USA).

**2.10 RNA-seq data analysis**

RNA-seq data analysis was completed by Gene Denovo Biotechnology Co.. In brief, the fastp^6^ was used to remove adapters or low-quality reads. After alignment of the paired-end reads to GRCH38 by HISAT2.2.4^3^ with default parameters, gene expression abundance was quantified to fragments per kilobase of exon model per million mapped reads (FPKM) using RSEM software.^7^ DESeq2 software^8^ was used to analyze the differential expression (FDR<0.05, |log_2_FC|>1). GO and KEGG enrichment analyses of differential genes were performed by Gene Denovo Biotechnology Co. (Guangzhou, China).

**2.11 Ribo-seq data analysis**

Ribo-seq data analysis was carried out by Gene Denovo Biotechnology Co. (Guangzhou, China). Briefly, low-quality reads and raw reads containing over 10% N bases were removed. Adapter sequences were trimmed. Reads between 20–40 bp in length were used for subsequent analysis. Ribosomal RNA, transfer RNAs, small nuclear RNAs and miRNAs were removed by mapping reads to the ribosome RNA database, GenBank and the Rfam database using Bowtie2.^9^ After alignment of the paired-end reads to the GRCH38 by STAR^10^ with 2-pass setting enabled, the reads mapped to noncoding RNAs were removed. The riboWaltz R package^11^ was used to plot the three-nucleotide periodicity. RSEM^7^ software was used to calculate the counts of reads in open reading frames of coding genes. The FPKM method was used to normalize the levels of gene expression. DESeq2 software^8^ was used to analyze the differential expression (FDR<0.05, |log2FC|>1). Gene Set Enrichment Analysis (GSEA) software^12^ and MSigDB were used for GSEA enrichment analysis.

Translation efficiency refers to the ratio of mRNA being translated to total mRNA. GO enrichment analyzed translational efficiency fold change greater than 2 of genes. To avoid the loss of some key developmental regulatory genes before and after differentiation, we assigned a minimum value of 0.001 to the Ribo-seq data and RNA-seq data of FPKM =NA before and after differentiation. To study the potential link between the changes in translational efficiency and the regulatory network that controls gene expression, genes were classified into five groups: Transcription: only genes with significant transcriptome difference. TE: only genes with significant differences in translation efficiency. Homedirection: there are significant differences in both dimensions, and the upward and downward directions are the same. Opposite: there are significant differences in both dimensions, and the upward and downward directions are opposite. Unchanged: there is no significant difference between the two dimensions.

**References**

1. Li X, Hu X, Tian GG, et al. C89 Induces Autophagy of Female Germline Stem Cells via Inhibition of the PI3K-Akt Pathway In Vitro. Cells 2019;8(6): 606.

2. Zhao H, Ge J, Wei J, et al. Effect of FSH on E(2)/GPR30-mediated mouse oocyte maturation in vitro. Cell Signal 2020;66: 109464.

3. Kim D, Langmead B, Salzberg SL. HISAT: a fast spliced aligner with low memory requirements. Nat Methods 2015;12(4): 357-60.

4. Zhang Y, Liu T, Meyer CA, et al. Model-based analysis of ChIP-Seq (MACS). Genome Biol 2008;9(9): R137.

5. Shen L, Shao NY, Liu X, et al. diffReps: detecting differential chromatin modification sites from ChIP-seq data with biological replicates. PLoS One 2013;8(6): e65598.

6. Chen S, Zhou Y, Chen Y, et al. fastp: an ultra-fast all-in-one FASTQ preprocessor. Bioinformatics 2018;34(17): i884-i890.

7. Li B, Dewey CN. RSEM: accurate transcript quantification from RNA-Seq data with or without a reference genome. BMC Bioinform 2011;12: 323.

8. Love MI, Huber W, Anders S. Moderated estimation of fold change and dispersion for RNA-seq data with DESeq2. Genome Biol 2014;15(12): 550.

9. Langmead B, Salzberg SL. Fast gapped-read alignment with Bowtie 2. Nat Methods 2012;9(4): 357-9.

10. Dobin A, Davis CA, Schlesinger F, et al. STAR: ultrafast universal RNA-seq aligner. Bioinformatics 2013;29(1): 15-21.

11. Lauria F, Tebaldi T, Bernabo P, et al. riboWaltz: Optimization of ribosome P-site positioning in ribosome profiling data. PLoS Comput Biol 2018;14(8): e1006169.

12. Subramanian A, Tamayo P, Mootha VK, et al. Gene set enrichment analysis: a knowledge-based approach for interpreting genome-wide expression profiles. Proc Natl Acad Sci U S A 2005;102(43): 15545-50.
