# Supplementary figures and images for: NAT10‐mediated ac4C modification promotes ectoderm differentiation of human embryonic stem cells via acetylating NR2F1 mRNA
Source: Cell Prolif. 2023 Dec 2;57(4):e13577. doi: 10.1111/cpr.13577 (PMC10984107; doi:10.1111/cpr.13577)

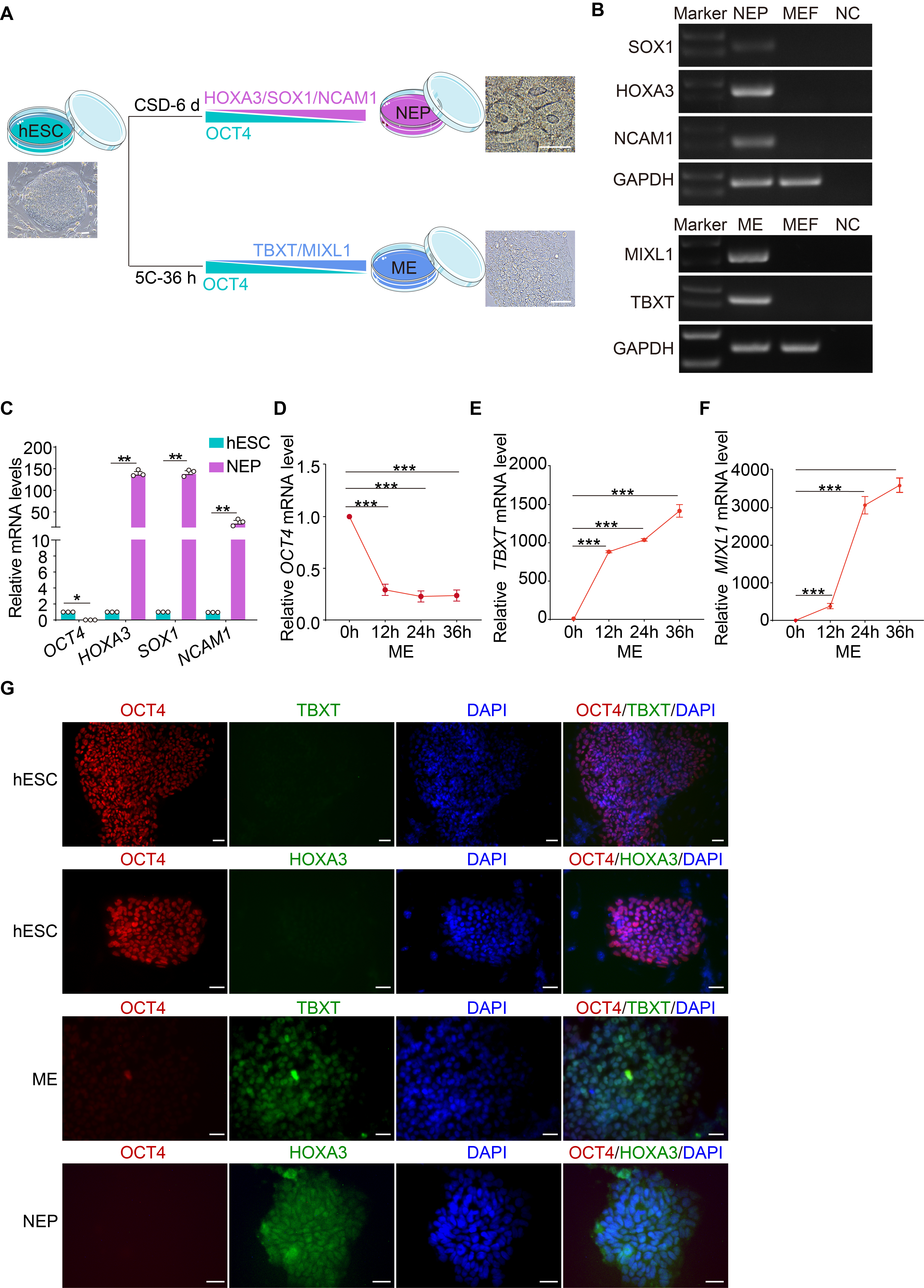

Supplement: Supplementary file 2 — FIGURE S1. Identification of hESC‐derived NEP cells and ME cells. (A) A scheme for the differentiation of hESCs to ME cells and NEP cells. S: SB431542; C: CHIR99021; D: DMH1; Representative photos at the indicated stages are shown. Scale bar = 50 μm. (B) RT‐PCR analysis of SOX1, HOXA3, NCAM1 in NEP cells and TBXT and MIXL1 expression in ME cells. GAPDH served as an endogenous normalizer. (C) qRT‐PCR analysis of OCT4, SOX1, HOXA3 and NCAM1 mRNA levels in NEP cells at day 7 and hESCs (normalised to GAPDH). (D‐F) qRT‐PCR analysis of OCT4 (D), TBXT (E) and MIXL1 (F) at different times (0 h, 12 h, 24 h and 36 h) during the differentiation of hESCs into ME cells. (G) Immunofluorescence assay was carried out using the anti‐OCT4 antibody (red), the anti‐HOXA3 antibody (green) and the anti‐TBXT antibody (green) on hESCs, the anti‐HOXA3 antibody (green), the anti‐OCT4 antibody (red) on NEP cells and anti‐TBXT antibody (green), the anti‐OCT4 antibody (red) on ME cells. DAPI was used to label the nuclei (blue). Scale bar = 50 μm. Data display the mean ± SEM. [file CPR-57-e13577-s003.jpg]

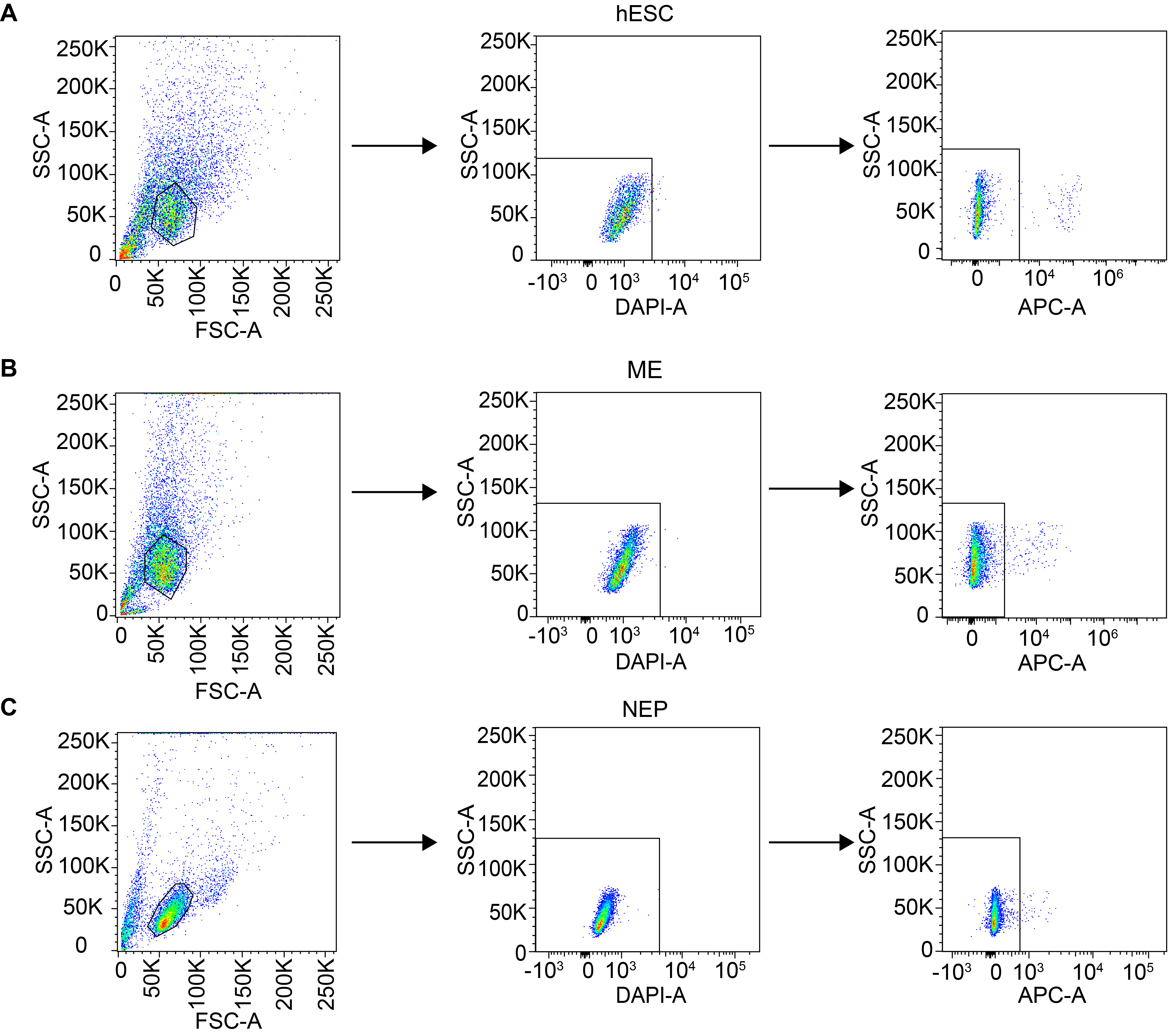

Supplement: Supplementary file 3 — FIGURE S2. Gating strategy for cell purification. (A–C) After gating by SSC and FSC, DAPI was used to exclude dead cells. Then CD29 was used to exclude mouse feeder cells. The remaining live hESCs (A), ME cells (B) and NEP cells (C) were retrieved for further analysis. SSC: side scatter. FSC: forward scatter. [file CPR-57-e13577-s010.jpg]

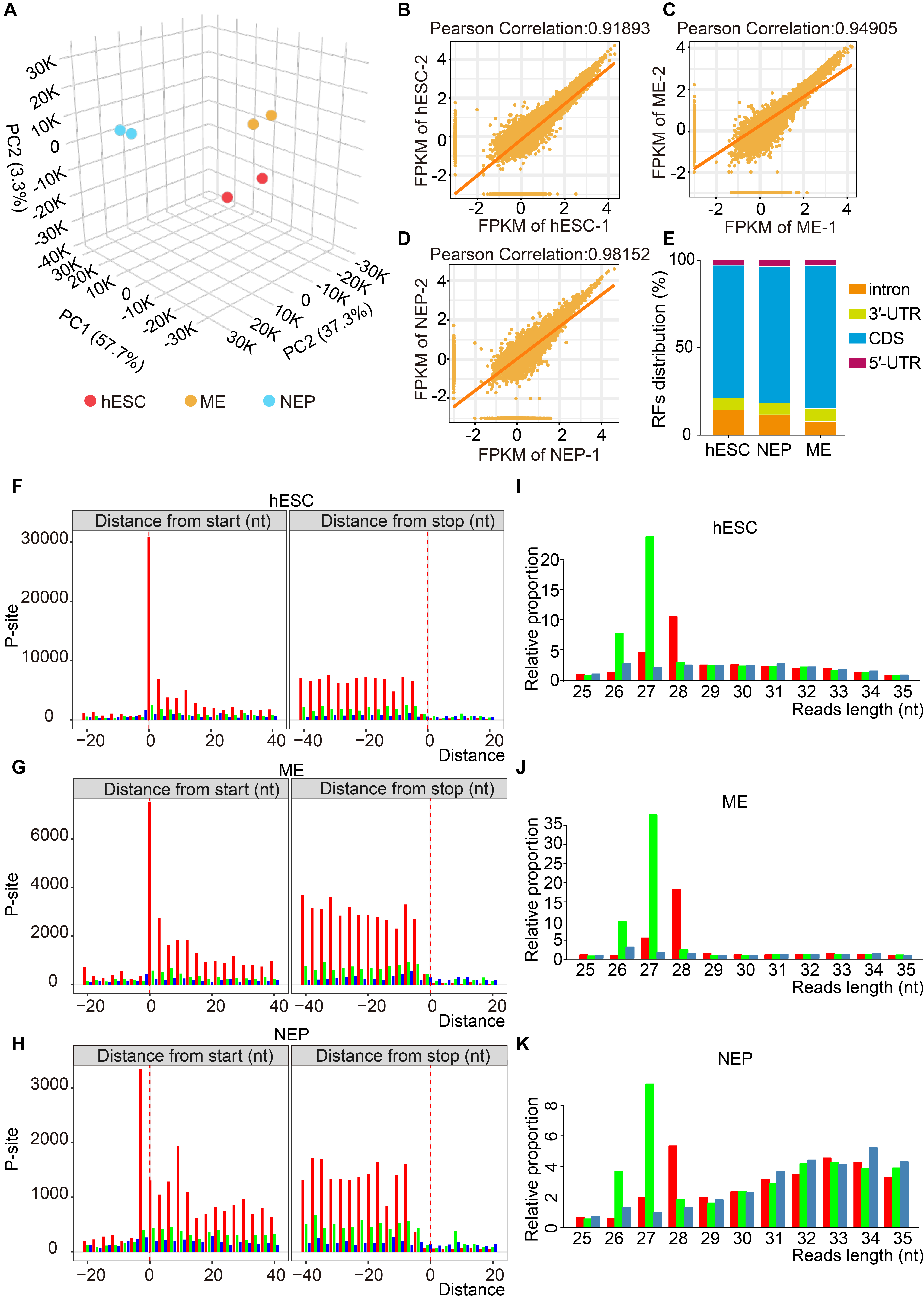

Supplement: Supplementary file 4 — FIGURE S3. Quality control of Ribo‐seq data. (A) Three‐dimensional PCA plot of hESCs and hESC‐derived NEP cells and ME cells. (B–D) Repetitive scatter plots were used to evaluate the repeatability of hESCs (B), ME cells (C) and NEP cells (D) within the group. (E) Distribution of ribosome footprints (RFs) in coding genes. (F–H) Distribution of the abundance of all ribosome footprints (RFs) in hESCs (F), ME (G) and NEP cells (H) around the start and end codons. (I–K) Bar plots displaying the distribution of codons for RFs of each length in hESCs (I), ME (J) and NEP cells (K). [file CPR-57-e13577-s005.jpg]

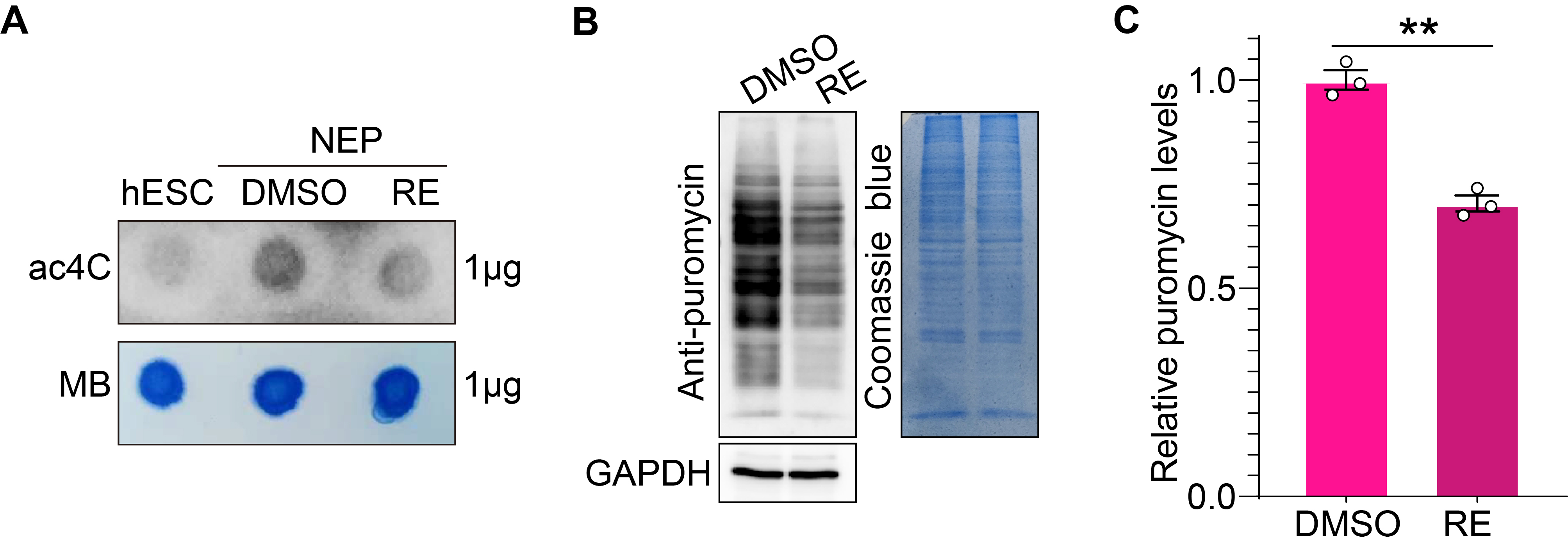

Supplement: Supplementary file 5 — FIGURE S4. The decrease in ac4C modification levels leaded to a decrease in the synthesis of new proteins. (A) mRNA dot blot analysis the expression levels of ac4C modification in hESCs, NEP cells and RE‐treated NEP cells. (B) Global translation of DMSO‐treated NEP cells and RE‐treated NEP cells (5 μM). Coomassie blue and GAPDH were used as control. RE: remodelin hydrobromide. (C) Quantitation of relative puromycin levels. [file CPR-57-e13577-s011.jpg]

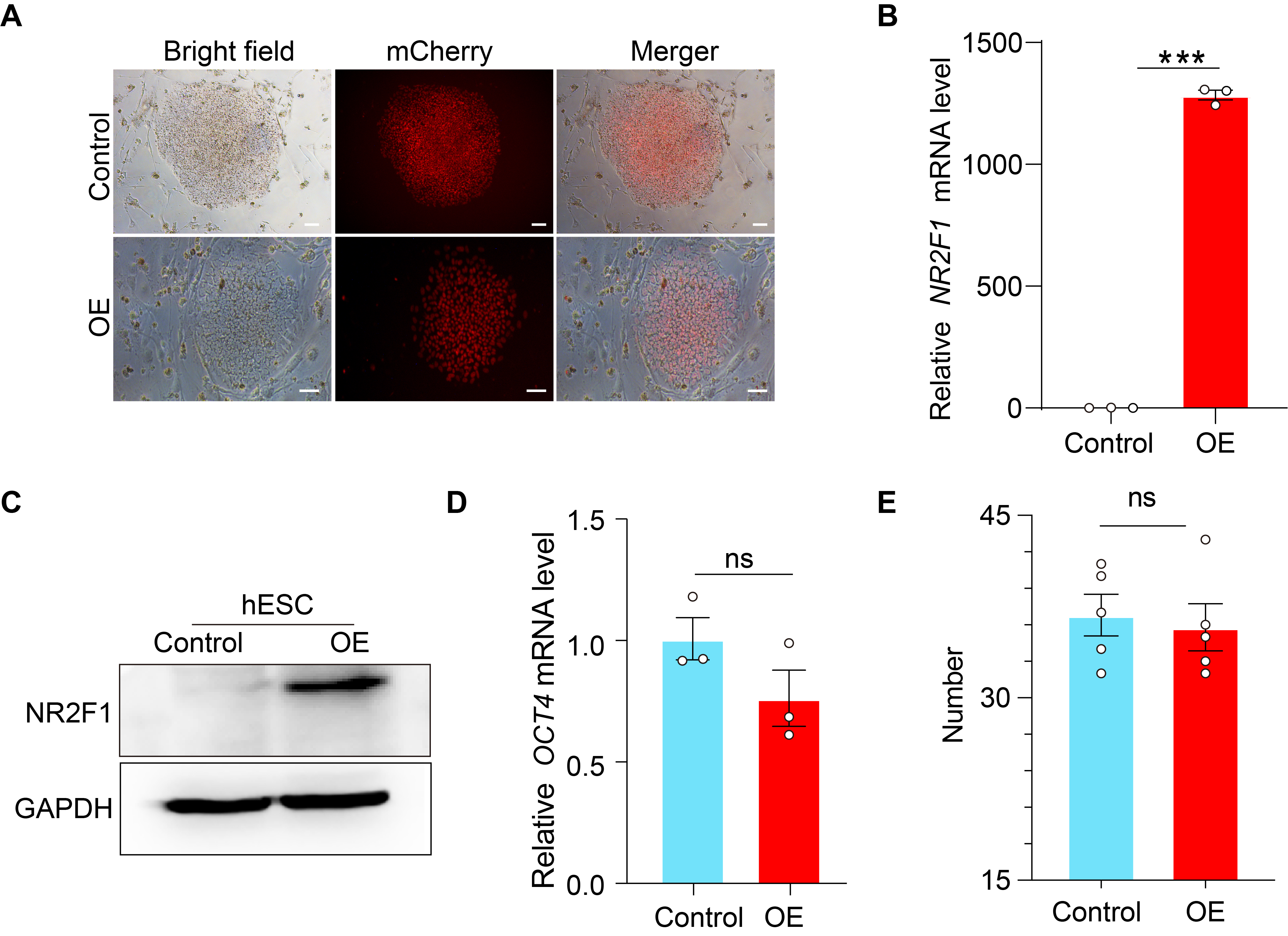

Supplement: Supplementary file 6 — FIGURE S5. NR2F1 is not the necessary factor for self‐renewal of hESCs. (A) Bright field pictures of OE‐control hESCs and OE‐NR2F1 hESCs. (B, C) NR2F1 mRNA (B) and protein (C) levels in control and OE‐NR2F1 human ESCs. Error bars display mean ± SEM. (D) The expression levels of OCT4 mRNA were tested used the qRT‐PCR method. (E) The number of clones was statistically analysed in control group and OE‐NR2F1 group. [file CPR-57-e13577-s002.jpg]

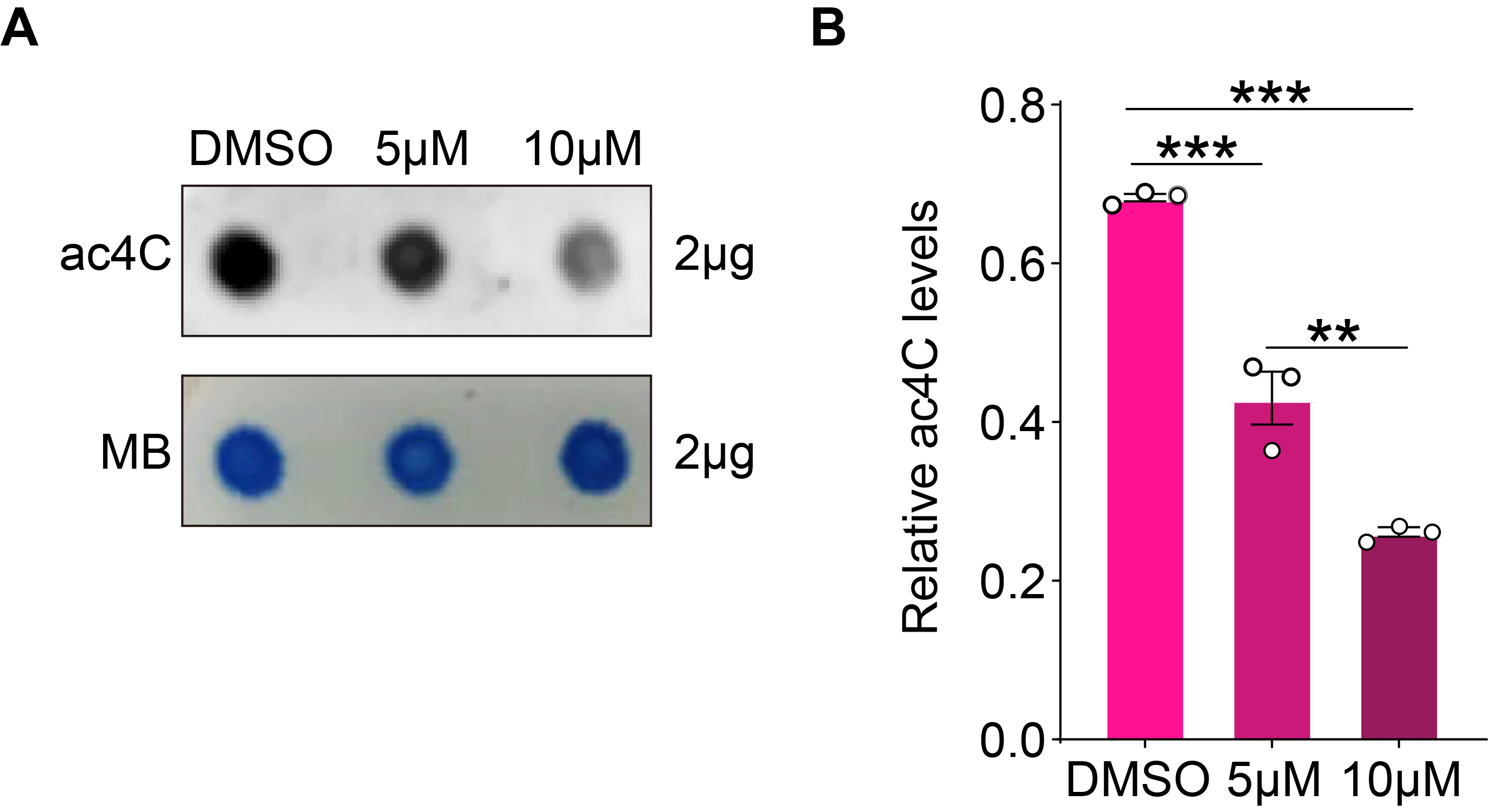

Supplement: Supplementary file 7 — FIGURE S6. Remodelin hydrobromide induced the decrease of ac4C modification. (A) mRNA dot blot analysis the ac4C modification levels in hESCs with different doses of RE for 2 days. RE: remodelin hydrobromide. (B) Quantitation of relative ac4C levels in (A) (n = 3). [file CPR-57-e13577-s008.jpg]
